# Metabolite-related dietary patterns and the development of islet autoimmunity

**DOI:** 10.1038/s41598-019-51251-4

**Published:** 2019-10-15

**Authors:** Randi K. Johnson, Lauren Vanderlinden, Brian C. DeFelice, Katerina Kechris, Ulla Uusitalo, Oliver Fiehn, Marci Sontag, Tessa Crume, Andreas Beyerlein, Åke Lernmark, Jorma Toppari, Anette-G. Ziegler, Jin-Xiong She, William Hagopian, Marian Rewers, Beena Akolkar, Jeffrey Krischer, Suvi M. Virtanen, Jill M. Norris, The TEDDY Study Group, Kimberly Bautista, Judith Baxter, Daniel Felipe-Morales, Kimberly Driscoll, Brigitte I. Frohnert, Marisa Gallant, Patricia Gesualdo, Michelle Hoffman, Rachel Karban, Edwin Liu, Andrea Steck, Kathleen Waugh, Olli G. Simell, Annika Adamsson, Suvi Ahonen, Mari Åkerlund, Anne Hekkala, Henna Holappa, Heikki Hyöty, Anni Ikonen, Jorma Ilonen, Sinikka Jäminki, Sanna Jokipuu, Leena Karlsson, Miia Kähönen, Mikael Knip, Minna-Liisa Koivikko, Mirva Koreasalo, Kalle Kurppa, Jarita Kytölä, Tiina Latva-aho, Katri Lindfors, Maria Lönnrot, Elina Mäntymäki, Markus Mattila, Katja Multasuo, Teija Mykkänen, Tiina Niininen, Sari Niinistö, Mia Nyblom, Sami Oikarinen, Paula Ollikainen, Sirpa Pohjola, Petra Rajala, Jenna Rautanen, Anne Riikonen, Minna Romo, Suvi Ruohonen, Satu Simell, Maija Sjöberg, Aino Stenius, Päivi Tossavainen, Mari Vähä-Mäkilä, Sini Vainionpää, Eeva Varjonen, Riitta Veijola, Irene Viinikangas, Desmond Schatz, Diane Hopkins, Leigh Steed, Jennifer Bryant, Katherine Silvis, Michael Haller, Melissa Gardiner, Richard McIndoe, Ashok Sharma, Stephen W. Anderson, Laura Jacobsen, John Marks, P. D. Towe, Ezio Bonifacio, Miryam D’Angelo, Anita Gavrisan, Cigdem Gezginci, Anja Heublein, Verena Hoffmann, Sandra Hummel, Andrea Keimer, Annette Knopff, Charlotte Koch, Sibylle Koletzko, Claudia Ramminger, Roswith Roth, Marlon Scholz, Joanna Stock, Katharina Warncke, Lorena Wendel, Christiane Winkler, Daniel Agardh, Carin Andrén Aronsson, Maria Ask, Jenny Bremer, Corrado Cilio, Emelie Ericson-Hallström, Annika Fors, Lina Fransson, Thomas Gard, Rasmus Bennet, Monika Hansen, Susanne Hyberg, Hanna Jisser, Fredrik Johansen, Berglind Jonsdottir, Silvija Jovic, Helena Elding Larsson, Marielle Lindström, Markus Lundgren, Maria Månsson-Martinez, Maria Markan, Jessica Melin, Zeliha Mestan, Caroline Nilsson, Karin Ottosson, Kobra Rahmati, Anita Ramelius, Falastin Salami, Anette Sjöberg, Birgitta Sjöberg, Carina Törn, Anne Wallin, Åsa Wimar, Sofie Åberg, Michael Killian, Claire Cowen Crouch, Jennifer Skidmore, Ashley Akramoff, Masumeh Chavoshi, Kayleen Dunson, Rachel Hervey, Rachel Lyons, Arlene Meyer, Denise Mulenga, Jared Radtke, Matei Romancik, Davey Schmitt, Julie Schwabe, Sarah Zink, Sarah Austin-Gonzalez, Maryouri Avendano, Sandra Baethke, Rasheedah Brown, Brant Burkhardt, Martha Butterworth, Joanna Clasen, David Cuthbertson, Christopher Eberhard, Steven Fiske, Jennifer Garmeson, Veena Gowda, Kathleen Heyman, Belinda Hsiao, Christina Karges, Francisco Perez Laras, Hye-Seung Lee, Qian Li, Shu Liu, Xiang Liu, Kristian Lynch, Colleen Maguire, Jamie Malloy, Cristina McCarthy, Aubrie Merrell, Steven Meulemans, Hemang Parikh, Ryan Quigley, Cassandra Remedios, Chris Shaffer, Laura Smith, Susan Smith, Noah Sulman, Roy Tamura, Dena Tewey, Michael Toth, Kendra Vehik, Ponni Vijayakandipan, Keith Wood, Jimin Yang, Liping Yu, Dongmei Miao, Polly Bingley, Alistair Williams, Kyla Chandler, Olivia Ball, Ilana Kelland, Sian Grace, Ben Gillard, Masumeh Chavoshi, Jared Radtke, Julie Schwabe, Bill Wikoff, Dmitry Grapov, Tobias Kind, Mine Palazoglu, Luis Valdiviez, Benjamin Wancewicz, Gert Wohlgemuth, Joyce Wong, Sandra Ke, Niveen Mulholland, Kasia Bourcier, Thomas Briese, Suzanne Bennett Johnson, Eric Triplett

**Affiliations:** 1 Department of Epidemiology, Colorado School of Public Health, University of Colorado Anschutz Medical Campus, Aurora, USA; 2 Department of Biostatistics and Informatics, Colorado School of Public Health, University of Colorado Anschutz Medical Campus, Aurora, USA; 3 UC Davis Genome Center—Metabolomics, University of California Davis, Davis, USA; 4 Health Informatics Institute, University of South Florida, Tampa, USA; 5 Department of Molecular and Cellular Biology, University of California Davis, Davis, USA; 6 Institute of Computational Biology, Helmholtz Zentrum München, Neuherberg, Germany; 7 Institute of Diabetes Research, Helmholtz Zentrum München, and Klinikum rechts der Isar, Technische Universität München, and Forschergruppe Diabetes e.V., Neuherberg, Germany; 8 Department of Clinical Sciences, Lund University/CRC, Lund, Sweden; 9 Department of Pediatrics, Turku University Hospital, Turku, Finland; 10 Institute of Biomedicine, Research Centre for Integrated Physiology and Pharmacology, University of Turku, Turku, Finland; 11 Center for Biotechnology and Genomic Medicine, Augusta University, Augusta, USA; 12 Pacific Northwest Diabetes Institute, Seattle, USA; 13 Barbara Davis Center for Childhood Diabetes, University of Colorado Anschutz Medical Campus, Aurora, USA; 14 National Institutes of Diabetes and Digestive and Kidney Disorders, National Institutes of Health, Bethesda, USA; 15 National Institute for Health and Welfare, Tampere, Finland; 16 University of Tampere, Tampere, Finland; 17 Tampere University Hospital, Tampere, Finland; 18 University of Oulu, Oulo, Finland; 19 Turku University Hospital, Hospital District of Southwest Finland, Turku, Finland; 20 Oulu University Hospital, Oulo, Finland; 21 University of Eastern Finland Kuopio, Kuopio, Finland; 22 University of Florida, Gainesville, FL USA; 23 Pediatric Endocrine Associates, Children’s Healthcare of Atlanta, Atlanta, GA USA; 24 Center for Regenerative Therapies, TU Dresden, Dresden, Germany; 25 Dr. von Hauner Children’s Hospital, Department of Gastroenterology, Ludwig Maximillians University Munich, München, Germany; 26 University of Bonn, Department of Nutritional Epidemiology, Bonn, Germany; 27 Bristol Medical School, University of Bristol, Bristol, UK; 28 NIDDK Biosample Repository at Fisher BioServices, Rockville, MD USA; 29 National Institutes of Allergy and Infectious Diseases, North Bethesda, MD USA; 30 Columbia University, New York, NY USA; 31 Florida State University College of Medicine, Tallahassee, FL USA

**Keywords:** Type 1 diabetes, Epidemiology

## Abstract

The role of diet in type 1 diabetes development is poorly understood. Metabolites, which reflect dietary response, may help elucidate this role. We explored metabolomics and lipidomics differences between 352 cases of islet autoimmunity (IA) and controls in the TEDDY (The Environmental Determinants of Diabetes in the Young) study. We created dietary patterns reflecting pre-IA metabolite differences between groups and examined their association with IA. Secondary outcomes included IA cases positive for multiple autoantibodies (mAb+). The association of 853 plasma metabolites with outcomes was tested at seroconversion to IA, just prior to seroconversion, and during infancy. Key compounds in enriched metabolite sets were used to create dietary patterns reflecting metabolite composition, which were then tested for association with outcomes in the nested case-control subset and the full TEDDY cohort. Unsaturated phosphatidylcholines, sphingomyelins, phosphatidylethanolamines, glucosylceramides, and phospholipid ethers in infancy were inversely associated with mAb+ risk, while dicarboxylic acids were associated with an increased risk. An infancy dietary pattern representing higher levels of unsaturated phosphatidylcholines and phospholipid ethers, and lower sphingomyelins was protective for mAb+ in the nested case-control study only. Characterization of this high-risk infant metabolomics profile may help shape the future of early diagnosis or prevention efforts.

## Introduction

Type 1 diabetes affects over 500,000 children globally, making it one of the most common metabolic illnesses in children^1^. Autoimmune destruction of the insulin-producing beta cells in the pancreas results in hyperglycemia and lifelong insulin dependency. Genetic risk factors are well described and likely interact with non-genetic risk factors to influence disease progression, though exact pathogenesis remains unclear^2^. The appearance of autoantibodies can be detected as early as 3 months of age and defines the beginning of islet autoimmunity (IA), the preclinical stage of the disease^3^. Efforts to better characterize metabolic dysregulation around the time of seroconversion and prior to the detection of autoantibodies may allow earlier identification of at-risk children and better understanding of the processes involved.

Metabolites reflect the interaction of numerous biological factors, including many that may influence the development of autoimmune diabetes, such as genetics, microbiome, and dietary intake. Metabolomics differences between IA cases and controls mostly have been found at the time of seroconversion, but are inconsistent across studies conducted in country-specific populations^4–7^. Previous country-specific studies used different laboratories to measure varying types (primary/polar^5^, lipids^4,7–9^, both^6,10^) and amounts of metabolomics features (from 106^7^ to 540^6^), and conducted studies at varying ages and stages of the disease course (cord blood^4,7,8^, at seroconversion to IA^6^, longitudinally^5,9,10^). Given these methodological differences, it is unclear whether differences in study findings are due to technical artefacts, or whether they represent truly different associations by geography or other meaningful characteristic. The identification of generalizable metabolic profiles related to the development of early stages of the disease across several populations is important and may inform dietary interventions to prevent type 1 diabetes, which have so far proven unsuccessful^11^.

Traditional investigation of diet in the development of type 1 diabetes has examined effects of individual foods or food groups and nutrients such as cow’s milk^11–13^, fatty acids^14–18^, or vitamin D^19–22^. However, these approaches do not account for the complexity of the diet—the effects of single nutrients and foods are often too small to identify, or too highly correlated to be separated from each other^23^. Examining combinations of foods and metabolites may better elucidate the role of diet in IA, as it can account for synergistic or antagonistic effects of foods or nutrients contained in the diet, and differences in how they are processed in the body.

We aimed to identify metabolite-related dietary patterns associated with IA in the multinational The Environmental Determinants of Diabetes in the Young (TEDDY) study. We conducted a metabolome- and lipidome-wide association study to better characterize plasma metabolites and lipids distinguishing cases and controls both at the time of the first autoantibody detection, and prior to its development. We created dietary patterns summarizing candidate metabolites identified pre-IA, and tested the longitudinal association of those metabolite-related dietary patterns with the development of IA.

## Methods

### TEDDY study design

TEDDY is an international consortium that enrolled 8,676 newborn infants with a high- or moderate-risk class II HLA genotype between 2004 and 2010^24^. Participants are closely followed for the development of IA or type 1 diabetes, with study visits every three months from birth to age 48 months, and every three or six months thereafter depending on autoantibody status until the age of 15 years. Participating study centers include: Georgia/Florida, Colorado, and Washington in the U.S., and Finland, Sweden, and Germany in Europe. IA cases are defined by confirmed autoantibody positivity to either insulin (IAA), GAD (GADA), or IA-2 (IA-2A) on two consecutive study samples, the first of which defines the case’s event age.

A nested case-control biomarker study was designed using risk set sampling to select three controls per IA case (n = 418) that had developed in TEDDY as of May 2012. Eligible controls were autoantibody-negative at the case’s event age, and further matched on clinical center, sex, and family history of type 1 diabetes as previously described^25^. Secondary outcomes included cases positive for IAA only or GADA only at IA event time. IA-2A was excluded as an outcome since very few cases developed IA2 as their first and only persistent confirmed autoantibody at IA case-time. Multiple autoantibody positivity (mAb+) was defined as any subject positive for more than one autoantibody at IA event time, or who developed more than one autoantibody during follow-up.

The study methods have been carried out in accordance with the approved guidelines by local Institutional Review or Ethics Boards, including: Colorado Multiple Institutional Review Board (#04-0361); Medical College of Georgia Human Assurance Committee (2004–2010)/Georgia Health Sciences University Human Assurance Committee (2011–2012)/Georgia Regents University Institutional Review Board (2013–2017)/Augusta University Institutional Review Board (2017-present) (#HAC 0405380); University of Florida Health Center Institutional Review Board (#IRB201600277); Washington State Institutional Review Board (2004–2012)/Western Institutional Review Board (2013–present) (#20130211); Ethics Committee of the Hospital District of Southwest Finland (#Dnro168/2004); Bayerischen Landesärztekammer (Bavarian Medical Association) Ethics Committee (#04089); and Regional Ethics Board in Lund, Section 2 (2004–2012)/Lund University Committee for Continuing Ethical Review (2013-present) (#217/2004). The study is monitored by an External Evaluation Committee formed by the National Institutes of Health. Written informed consents were obtained from a parent and/or legal guardian for all participating children. Data described in the manuscript and code book will be made available upon request from the NIDDK Central Repository at https://www.niddkrepository.org/studies/teddy.

### Metabolomics data pre-processing

Metabolomics abundance measures (metabolites and lipids) were obtained for all cases and controls for each available study visit from birth until the case event time. Primary metabolites and complex lipids were quantified from citrate plasma using GC-TOF MS and CSH-QTOF MS data acquisition, respectively, at the NIH West Coast Metabolomics Center at the University of California, Davis. GC-TOF MS data were acquired as previously described^26^, with data processing and compound identification using the BinBase algorithm^27^, GC-TOF data were sum normalized followed by LOESS (locally weighted scatterplot smoothing) normalization. For complex lipids, samples were extracted by methyl-tert-butyl ether/methanol/water^28^, followed by chromatogram peak detection and alignments using Mass Profiler Professional (Agilent, Santa Clara, CA). Peaks detected in a minimum of 30% of samples were identified and quantification back-filled using the Fiehn laboratory’s LipidBlast spectral library, as previously described^29^. LOESS followed by batch ratio (QC samples were used to adjust sample batch median to global study median) normalization was performed across all the samples to estimate and remove analytical variance.

Prior to transformation, data quality checks included evaluation at the metabolite- and sample-level (Supplemental Fig. 1). Metabolites that were not detected in more than 10% of samples (6 metabolites), or with a coefficient of variation greater than or equal to 100% (286 metabolites) were excluded from further analyses. Samples with missing or zero values in greater than 10% of metabolomics features (n = 5) or with values more extreme than 4 standard deviations above or below the mean in greater than 30% of metabolomics features (n = 6) were removed from analyses. A total of 853 metabolites and lipids and 11,556 samples passed the quality checks. All metabolites were transformed using Box-Cox transformation analysis, and scaled^30^.

### Dietary intake and food groupings

Dietary assessment was carried out by 24-hour recall at the first clinic visit at 3–4.5 months of age, then by 3-day food record every 3 months until 12 months of age, and then every 6 months thereafter. TEDDY research staff provided detailed instruction and examples to families regarding completion of food records, as previously described^31^. From quantities of foods and dishes consumed, the amounts of energy and single foods contained therein were computed using in-house food record processing programs and food composition databases unique to each country^32^. The foods and dishes (e.g. wheat bread, apple-oat meal) consumed were quantified into main food groups (ie: cereals, fruits and berries, etc.) and subgroups (ie: wheat, rice, oats, citrus fruits, apple, berries, etc.) in grams per day (g/day) of intake. After quantification, the three food records were averaged to calculate the mean energy and food intake for each study subject on each study visit. Results of detailed harmonization studies of these country-specific food composition databases documented that the energy values and food subgroups used in this study were comparable across the TEDDY countries^32^.

For any food record where a subject was indicated as breast fed, we estimated the amount of breastmilk consumption using an algorithm developed by the Institute of Medicine^33^. First, we calculated the estimated energy requirement based on age and weight. The difference in the estimated energy requirement and the mean energy reported on the food record from food and formula was attributed to breastmilk. We calculated the amount (grams) of breastmilk consumed to achieve that energy intake using a conversion factor of energy density per 100 g, as follows: 65.3 kcal/100 g in Finland, 69 kcal/100 g in Germany, 68 kcal/100 g in Sweden, and 70 kcal/100 g in the U.S.

### Statistical analyses

**Figure 1:**
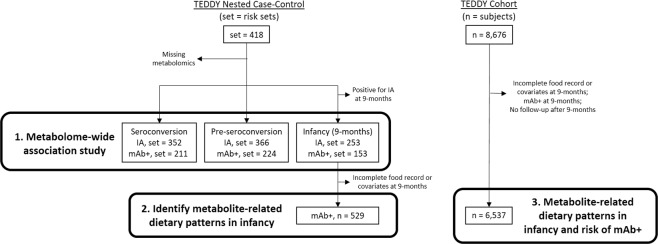
Data flow diagram summarizing selection and size of analysis population for all aims (numbered). From a nested case-control study in TEDDY, we tested the association of 853 metabolites with outcomes at the time of seroconversion to IA (sets = 352), the last sample prior to IA (sets = 366), and at infancy (sets = 253). We created dietary patterns explaining candidate mAb+ metabolites identified in infancy, when children (n = 529) were 9-months of age and autoantibody negative. All subjects with food records at 9-months in the full TEDDY cohort (n = 6,537) were scored on the dietary pattern, and the association with development of mAb+ tested. IA = islet autoimmunity, mAb+ = multiple autoantibody positive, sets = number of risk sets or matched strata, n = number of subjects.

Statistical analyses are described by aim below. First, we identified plasma metabolites and lipids associated with IA at three different time points, using a metabolome-wide association approach in TEDDY’s nested case-control study. Second, we created dietary patterns summarizing candidate metabolites identified in infancy. Finally, we tested the longitudinal association of infancy metabolite-related dietary patterns with development of mAb+ in the full TEDDY cohort. Figure 1 summarizes the population and data flow for all aims and analyses.

#### Metabolites associated with IA

Conditional logistic regression was used to calculate odds ratios (ORs) for the association of each transformed metabolite with the development of IA, adjusting for high-risk HLA genotype (DR3-DQA1*05:01-DQB1*02:01/DR4-DQA1*03:01-DQB1*03:02 versus all other), and age at blood draw. Some TEDDY subjects follow a long-distance protocol, in which blood is drawn and shipped to clinical centers before being processed for biomarker identification. Since plasma primary GC-TOF MS metabolic profiles are less stable with centrifugation delay^34^, we required an additional match for the long distance protocol between case and control samples.

Metabolomics analyses were run in three cross-sections. First, we examined metabolite differences between IA cases and controls at the first detection of autoantibody positivity (seroconversion), defined as the first of the two consecutive autoantibody positive visits for cases. Then, to identify metabolites and lipids that may differentiate IA cases and controls prior to the detection of autoantibodies (pre-seroconversion) we selected the most recent IA-free visit for cases. Finally, since autoimmunity can begin very early in life and both metabolomics and dietary factors are strongly related to age, we identified metabolites distinguishing IA cases and controls prior to the appearance of autoantibodies in infancy. The “infancy” cross-section was defined as an IA-free visit at 9 months of age for cases. For controls, the visit corresponding to the case visit was selected for all cross-sections. Children positive for autoantibodies at 9-months (n = 48 IA cases) and their matched controls were excluded from the infancy cross-section (Fig. 1).

We tested the association of each metabolite with the secondary outcomes described above: IAA, GADA, and mAb+. We considered p-value < 0.05 significant since traditional approaches for multiple comparison correction may be too strict for the unusually highly correlated metabolomics data or inappropriate given the exploratory nature of our study aims^35^. SAS version 9.4 was used for these analyses.

We focused on pathway enrichment, given that metabolites may capture perturbations in many upstream biological systems thereby complicating interpretation of individual associations. ChemRICH forms non-overlapping groups of metabolites based on chemical similarity and ontology mapping^36^. It calculated a single p-value for each group, and identified the most significant metabolite in each group as the “key compound” (http://chemrich.fiehnlab.ucdavis.edu/). Inputs for the ChemRICH analyses included the nominal p-value and odds ratio from the individual conditional logistic regression models, and chemical structure information from well-characterized known metabolites and lipids (p = 315, see Supplemental Table 1).

#### Metabolite-related dietary patterns preceding the appearance of autoantibodies

Reduced rank regression (RRR) was used to identify dietary patterns reflecting metabolites associated with IA. RRR creates linear combinations of foods (dietary patterns) that explain the maximum covariation in a second set of intermediate response variables (metabolites)^37^, thereby capturing disease-related variation in the diet rather than general eating behaviors identified from other dietary pattern methods^38^. We focused dietary pattern analysis on the infancy cross-section, since it is prior to the beginning of the autoimmune process and all children were the same age. Conducting dietary pattern analyses with foods in young children of different ages could be problematic, since not all foods are able to be eaten at all ages.

Foods from the food record were combined into 43 subgroups based on nutrient content and culinary usage (Supplemental Table 2)^32^. Intake of various foods varies greatly at 9-months of age, leading to some foods having a large proportion of subjects with no reported intake. Therefore, we filtered out food subgroups with a high proportion of non-eaters, as a way to deal with inflated zero distributions, as is common in studies using RRR^39^. Food subgroups that were shown to be comparable across TEDDY countries (ie harmonized), and were eaten by at least 40% of subjects in infancy were included in the creation of dietary patterns. The resulting dietary patterns therefore reflect foods that are most commonly eaten at 9-months among TEDDY study participants. Food subgroups were standardized to the age-specific mean and standard deviation of all TEDDY food records for dietary pattern analyses. The key compound in each significantly enriched metabolite group (identified by ChemRICH) was used as RRR response variables.

The number of dietary patterns needed to best explain the variation in metabolites was selected using the van der Voet T2 statistic^40^. The loadings (or relative weights) of food groups on each dietary pattern and the partial correlation with metabolite response variables was used to interpret each dietary pattern.

#### Metabolite-related dietary patterns and risk of IA

Infancy metabolite-related dietary patterns were first tested in the nested case-control study using conditional logistic regression as described above. Then we applied them to the full TEDDY cohort at 9 months of age, using the food group loadings to generate one score per pattern for each subject with complete food records (n = 6,537). The dietary pattern score is a linear combination of food intakes using the reported intake of each food weighted by the factor loadings^37^. The score indicates how similar the reported dietary intake of a subject is to the dietary pattern—with higher scores indicating a diet similar to the pattern, and lower scores indicating a diet dissimilar to the pattern.

Cox proportional-hazards models were used to test the association of metabolite-related dietary pattern scores at 9-months on risk of mAb+, adjusting for clinical center, high-risk HLA genotype, family history of type 1 diabetes, total energy intake, and sex. Adjustment factors used in multivariable models were selected based on standard adjustments used in the TEDDY study^31,41^. Time-to-event analyses were performed to evaluate whether metabolite-related dietary pattern scores were associated with mAb+ by the age of 6 years. Cases included those selected for the nested case-control study plus any additional cases that developed by January 2018, when the prospective data collection was cut for analyses. Given that risk factors for IA may differ by age, we restricted follow up to 6 years in the TEDDY cohort to ensure the cohort analysis represented a similarly-aged case-population as the nested case-control study. For consistency with the nested case-control study, the time-to-event was defined as the time from birth to the appearance of the first persistent confirmed autoantibody among IA cases who developed a second persistent confirmed autoantibody at any point. Subjects without complete covariate information, those developing mAb+ at 9-months (n = 53), or who had no follow-up after 9-months (n = 243) were excluded from survival analysis (Fig. 1).

## Results

### Metabolic dysregulation apparent at seroconversion and infancy

From the nested case-control study, there were 352 matched sets with metabolomics measures for 1 case and at least 1 control at seroconversion (mean (SD) case-age = 722 (446) days), 366 sets at pre-seroconversion (mean (SD) = 625 (412) days), and 253 sets at the 9-month infancy visit (mean (SD) = 283 (14) days) (Table 1). For secondary outcomes, 49% of IA cases were positive for IAA, 32% for GADA, and 60% for mAb+. The distribution of secondary outcomes was consistent across the seroconversion, pre-seroconversion, and 9-month infancy cross-sections. The majority of IA cases were from Sweden (32%) and Finland (28%). IA cases were 55% male, 22% had a first degree relative, and 12% had their seroconversion blood-draw following TEDDY’s long distance protocol (Supplemental Table 3). The distribution of matched sets by matching factors (clinical center, sex, first-degree relative status, and long distance protocol) was similar in the secondary outcomes compared to primary IA. Approximately 9% of the pre-seroconversion case samples (n = 34) occurred during the 9-month infancy visit.

**Table 1:** Description of matched sets (1 case and 1, 2 or 3 controls) for metabolomics analyses by outcome and cross-section.

Cross-section	IA		IAA*			GADA*			mAb+*		
	n	Case-age^†^, mean (SD)	n	% of IA cases	Case-age, mean (SD)	n	% of IA cases	Case-age, mean (SD)	n	% of IA cases	Case-age, mean (SD)
Seroconversion	352	722 (446)	171	48.6	586 (370)	113	32.1	888 (509)	211	59.9	655 (365)
Pre-Seroconversion	366	625 (412)	180	49.2	505 (366)	116	31.7	786 (445)	224	61.2	541 (346)
Infancy 9-months	253	283 (14)	114	45.1	283 (14)	83	32.8	284 (16)	153	60.5	282 (14)

*Secondary outcomes defined as IAA or GADA as first-appearing and only autoantibody at IA case-time (mutually exclusive), while mAb+ indicates IA case developed more than 1 persistent confirmed Ab at any point during follow-up.

^†^Age at the time of metabolomics blood draw, in days.

n = Number of matched sets (each set has 1 case and 1, 2, or 3 controls).

**Figure 2:**
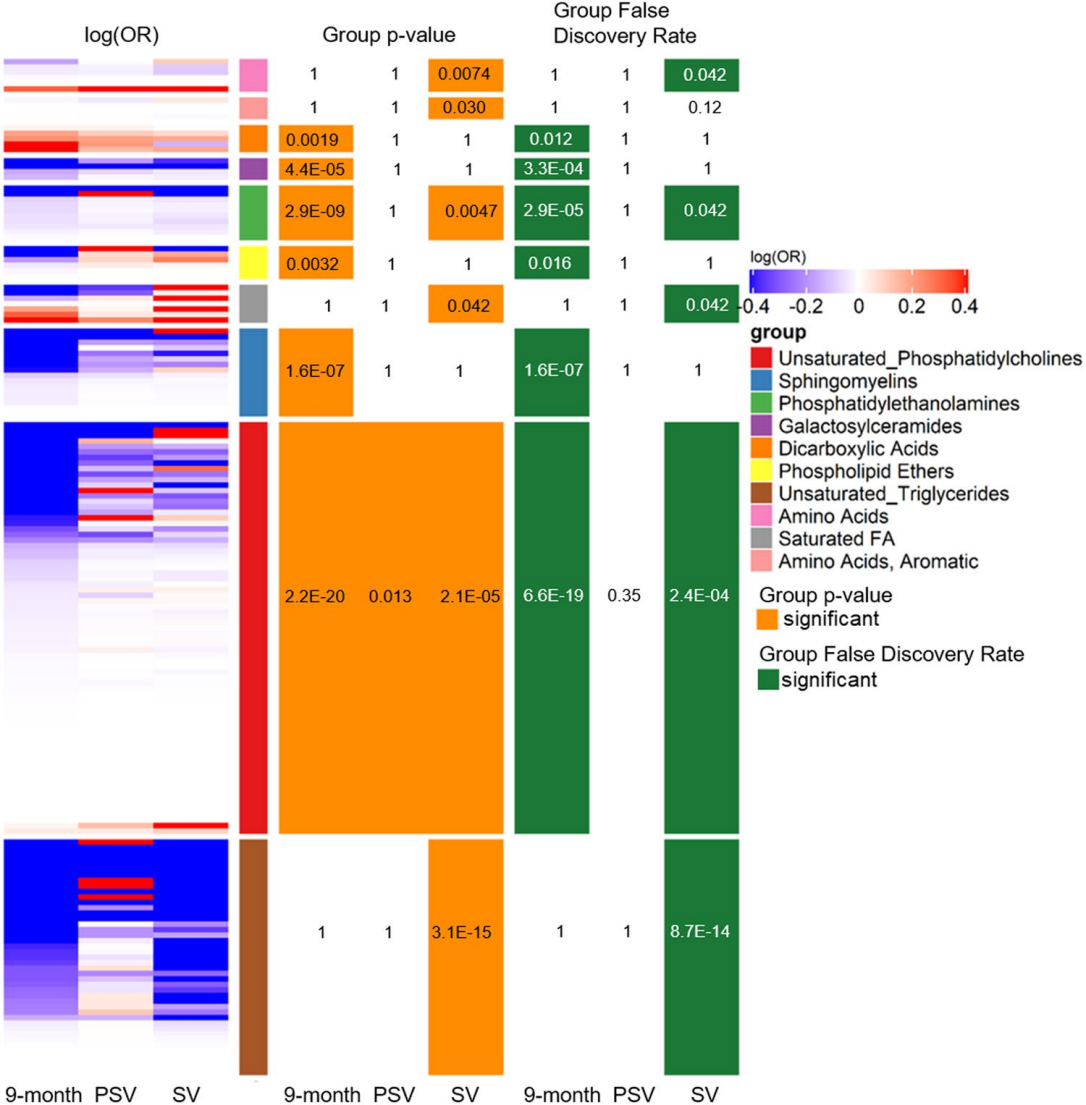
Chemically similar metabolite sets identified as significantly associated with mAb+ by ChemRICH. Each row is an individual metabolite, grouped by ChemRICH set and sorted by log(OR) within each set. Log(OR) > 0 (red) indicates a positive association between metabolite and mAb+, whereas log(OR) < 0 (blue) indicates an inverse association between metabolite and mAb+. Phosphatidylcholines were significantly lower in cases compared to controls in infancy (9-month), just prior to seroconversion (PSV), and at seroconversion to primary IA (SV). Other phospholipids were significantly lower in cases only in infancy, while other metabolite groups, such as unsaturated triglycerides and amino acids, distinguished cases and controls at seroconversion. Metabolite groups identified as significant (group p-value < 0.05) in any cross-section are shown, along with the corresponding adjusted p-value for the group (group false discovery rate).

Conditional logistic regression results from the metabolome-wide association study indicated metabolic dysregulation in cases compared to matched controls at seroconversion and during infancy (Supplemental Fig. 2). More metabolites and lipids were different by case status when restricting to the mAb+ outcome (p = 130, 15%) compared to the IA outcome (p = 64, 7.5%). There were few metabolites associated with the secondary outcomes IAA first and GADA first in any cross-section. Therefore, we focused the metabolomics set enrichment analyses on the mAb+ outcome, which represented 60% of IA cases and had the largest signal of metabolomics differences between cases and controls. ChemRICH identified seven groups of chemically similar metabolites that were significantly different among mAb+ cases and controls at seroconversion, one group at pre-seroconversion, and six groups in infancy (Fig. 2, Supplemental Table 4).

Of the metabolite groups identified as different between mAb+ cases and controls, only unsaturated phosphatidylcholines (PC) were consistently dysregulated in all three analyses (p-value for group in seroconversion = 2.1 × 10^−5^, pre-seroconversion = 0.013, infancy = 2.2 × 10^−20^), with the majority of the individual metabolites being lower in mAb+ cases compared with controls (OR < 1) (Fig. 2). Similarly, phosphatidylethanolamines (PE) were lower in mAb+ cases (OR < 1) at both seroconversion (p-value = 0.0047) and in infancy (p-value = 2.9 × 10^−6^).

Other than PCs and PEs, distinct metabolite groups distinguished mAb+ cases from their controls at the time of seroconversion to primary IA compared to during infancy prior to the appearance of any autoantibodies. At seroconversion, mAb+ cases had lower levels of unsaturated triglycerides (p-value = 3.1 × 10^−15^), amino acids (p-value = 0.0074), diglycerides (p-value = 0.014), and aromatic amino acids (p-value = 0.03), and higher levels of saturated fatty acids (p-value = 0.0074). In infancy, other phospholipids were significantly protective for mAb+ (majority of OR < 1), including sphingomyelins (SM, p-value = 1.1 × 10^−8^) and phospholipid ethers (EtherPL, p-value = 0.0032), along with the glucosylceramides (GlcCer, p-value = 4.4 × 10^−5^). Three dicarboxylic acids were significantly higher in mAb+ cases compared to controls in infancy (OR > 1, p-group = 0.0019).

### Infant metabolite-related dietary patterns and risk of mAb+

From each of the six groups identified by ChemRICH in infancy, we used the key metabolite (most significant one) as a response variable in dietary pattern analyses, including: PC (34:3), SM (d41:2) A, PE (34:2), GlcCer (d41:1), adipic acid, and PC (p-32:0) or PC (o-32:1) (EtherPL).

**Figure 3:**
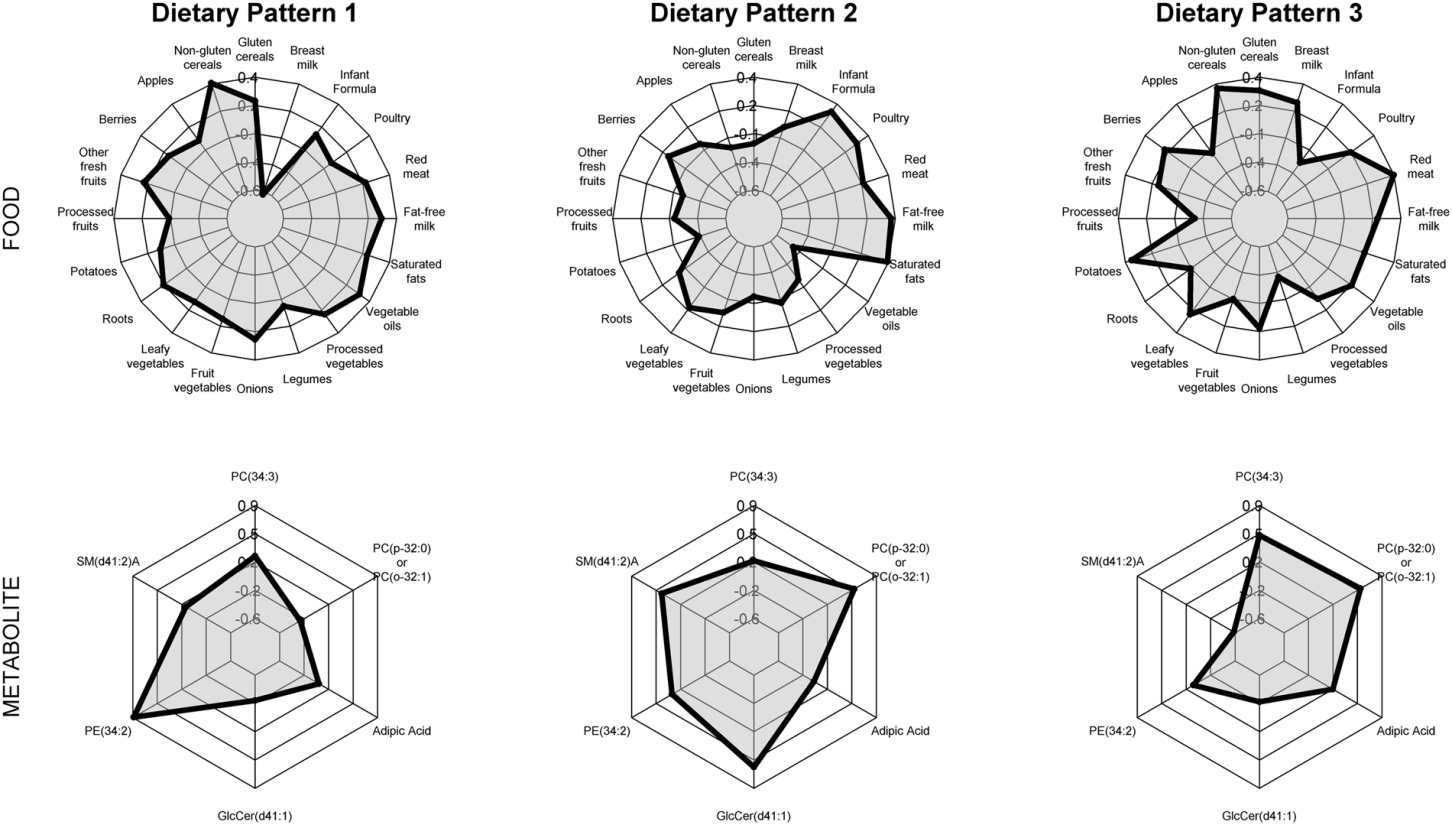
Food group loadings and metabolite weights for metabolite-related dietary patterns. In total, the three dietary patterns explained 8% of metabolite variation and 29.3% of food variation. For food, the radial axis indicates the loading on each dietary pattern (Range: −0.6 to 0.4), and is used to interpret which combinations of foods are influential in the dietary pattern. Similarly, the metabolite radial axis indicates the weight of each metabolite on each dietary pattern (Range: −0.6 to 0.9), indicating which combination of metabolites are explained by each dietary pattern. For example, subjects scoring high on dietary pattern 1 had diets higher in non-gluten containing cereals, onions, vegetable oils, and fat-free milk, and lower in breast milk. This diet corresponded to higher levels of PE (34:2).

Reduced rank regression identified three dietary patterns that explained 8% of the variation in metabolites and 29.3% of the food variation. Food groups factor loadings and metabolite variable weights used to interpret each dietary pattern are shown in Fig. 3. More extreme factor loadings or variable weights indicate the food or metabolite was influential in the dietary pattern. Infants scoring high on Dietary Pattern 1 ate more non-gluten containing cereals, onions, vegetable oils, and fat-free milk (positive factor loadings), and less breast milk (negative factor loadings). This diet corresponded to higher levels of PE (34:2), as indicated by the higher variable weight. Infants scoring high on Dietary Pattern 2 ate diets with higher saturated fats, fat-free milk, poultry, and infant formula, and lower in potatoes and vegetable oils. This diet corresponded to higher levels of SM (d41:2) A, GlcCer (d41:1), and PC (p-32:0) or PC (o-32:1). Finally, 9-month infants scoring high on Dietary Pattern 3 ate diets higher in breast milk, red meat, potatoes, and cereals, and lower in processed fruits, legumes, and infant formula. High scores on Dietary Pattern 3 corresponded to higher levels of PC (34:3) and PC (p-32:0) or PC (o-32:1), and lower levels of SM (d41:2) A. The correlation between metabolites and dietary patterns followed similar patterns (Supplemental Table 5).

Dietary patterns generated from metabolites and food intake in the nested case-control study were applied to the full cohort to generate one metabolite-related dietary pattern score for each dietary pattern on all 9-month diet records. Subjects developing mAb+ by age 6 years were more likely to have a first-degree relative (FDR) with type 1 diabetes and to have high-risk HLA-DR3/4 genotypes (Supplemental Table 6). Dietary Pattern 3 was significantly protectively associated with mAb+ in the nested case-control study (OR = 0.67, 95%CI = 0.48–0.94, Table 2). However, there was no association seen in time-to-event analyses applied to the whole cohort and adjusted for clinical center, sex, HLA-DR3/4, and FDR (HR = 0.98, 95%CI = 0.83–1.16, Table 2). No other 9-month metabolite-related dietary patterns were associated with development of mAb+ in the TEDDY cohort. Results did not change in a sensitivity analysis in which we adjusted for an additional 17 covariates that TEDDY has identified as associated with development of IA (race-ethnicity, maternal education, maternal age, introduction of probiotics before 28 days, introduction of probiotics at or after 28 days, weight for age z-score at 12 months, and number of minor alleles for rs2476601, rs2816316, rs11711054, rs10517086, rs4948088, rs1004446, rs7111341, rs2292239, rs3184504, rs3825932, rs12708716) (data not shown).

**Table 2:** Dietary patterns at 9-months of age associated with risk of mAb+ in TEDDY.

Metabolite-related dietary patterns	Nested Case-Control* n = 147 mAb+ cases			Cohort^†^ n = 300 mAb+ cases by 6 years		
	OR	95%CI		HR	95%CI	
1	0.85	0.68	1.05	0.95	0.83	1.08
2	0.81	0.61	1.08	0.89	0.78	1.02
3	0.67	0.48	0.96	0.98	0.83	1.16

*Conditional logistic regression models adjusted for age at metabolomics blood draw and total energy.

^†^Survival models adjusted for clinical center, sex, FDR, total energy, and HLA DR3/4.

OR = Odds Ratio, CI = confidence interval, HR = Hazard Ratio.

## Discussion

We identified dysregulated metabolism at the onset of and preceding stage 1 diabetes (mAb+) in a multi-national, prospective type 1 diabetes study. PC and PE metabolite groups were consistently decreased in mAb+ cases compared to controls both prior to and at the time of seroconversion. Unsaturated triglycerides and amino acid groups were lower among mAb+ cases at seroconversion only, whereas SM, GlcCer, and EtherPL lipids were lower among mAb+ cases in infancy only. While an infancy dietary pattern explaining choline- and sphingosine- containing lipids was associated with mAb+ in the nested case-control study, this association was not observed in the full TEDDY cohort.

Dicarboxylic acids were the only metabolite group we found associated with increased risk of mAb+ in infancy. Adipic acid was the key compound of the dicarboxylic acids group. Other dicarboxylic acids associated with increased mAb+ risk included the tricarboxylic acid (TCA) cycle intermediaries succinic acid and malic acid. While metabolomics studies in Norway and Germany did not identify TCA cycle metabolites^5,6^, a previous study in Finnish children found both succinic acid and glutamic acid were increased in type 1 diabetes cases 0–9 months prior to autoantibody appearance^10^. We were not able to examine glutamic acid as its measurement was inhibited by the use of citrate tubes for plasma collection and storage in TEDDY. Through their regulation of demethylase activity, succinic acid and other TCA cycle intermediaries may be important regulators of DNA and histone methylation^42^, which may have some links to type 1 diabetes pathogenesis^43^.

The remainder of metabolite groups distinguishing mAb+ cases from controls in infancy belonged to lipid classes, some of which have been inconsistently associated with type 1 diabetes endpoints. We identified phospholipid dysregulation of PC and PE metabolites prior to mAb+ in infancy and at the time of seroconversion. While Oresic *et al*.^10^ similarly found lower phosphatidylcholines in children who later developed type 1 diabetes, Pflueger *et al*. found higher levels of triglycerides and PUFA-containing phosphatidylcholines in autoantibody-positive children^6^. Unsurprisingly, the direction of association is different before and after the appearance of autoantibodies, as has been reported for other risk factors for type 1 diabetes, such as erythrocyte membrane fatty acid levels^15,17^ or diabetes susceptibility genes^44^.

Sphingolipid metabolism plays a role in diabetic pathologies, including regulating beta-cell apoptosis, proinsulin and insulin folding in the endoplasmic reticulum, and cytokine secretion^45^. The evidence supporting this connection has been recently extended from animal models into human islet cells^46^. We identified two sphingolipid groups as significantly lower in infancy for mAb+ cases versus controls, including the SM group, which were previously identified in type 1 diabetes metabolomics studies^9,10^, and the GlcCer group. As a whole, sphingolipids have been characterized as both pro- and anti-inflammatory. Endogenous sphingolipids are metabolically involved in T-cell regulation, autoimmunity, and inflammation^47^, yet consumption of dietary sphingolipids have been linked to anti-inflammatory responses^48^. The protective effects of dietary sphingolipids may operate via changes in gut microbiota or by activating other cofactors such as peroxisome proliferator-activated receptor γ expression^49^, both of which have been implicated in type 1 diabetes^50^. While outside the scope of this study, targeted characterization of the relationship between sphingolipid dietary intake and metabolite levels in future research might help to disentangle reported contrasting effects, which likely depend on other factors such as specific sphingolipid structure and existing metabolic state.

We identified choline-containing lipid groups (PC, SM, EtherPL) as protective for development of mAb+ at 9 months of age, consistent with previous studies conducted at birth and 3 months of age^7,9,10^. Choline is important for rapid growth and development in infancy, as a constituent of phospholipid cellular membranes. Additionally, it may play a role in insulin resistance or energy metabolism, perhaps through its role as a methyl donor for epigenetic changes^51^.

A metabolite-related dietary pattern reflecting choline-containing foods and metabolites was protective for mAb+ in the nested case-control study; however, no dietary pattern at 9-months was associated with development of mAb+ by age six years in the full TEDDY cohort. There are several factors that could contribute to the lack of dietary pattern association found. First, untargeted metabolomics and 3-day food records may not be measured precisely enough to successfully identify disease-related dietary patterns at such a young age where variability in both is large. Second, using the most significant metabolite in each group may not be the best choice of response variable, which is the variable that determines the ability of reduced rank regression to capture disease-related variation in the diet^52^. Metabolites may reflect other environmental factors, such as medication or microbiome, and therefore be poorly correlated with dietary intake. Metabolomics measures were further limited because they were quantified from non-fasting samples, which has been shown to differentially impact serum metabolic profiles related to dietary factors^53^.

We identified metabolic dysregulation prior to the detection of autoantibodies that distinguished children whose lifetime risk for symptomatic (Stage 3) type 1 diabetes approaches 100%^54^. Metabolomics differences were more apparent when comparing the high-risk mAb+ group to controls than comparing all cases of IA to controls. Few differences were identified by the type of first-appearing autoantibody. This metabolomics discovery was more comprehensive and generalizes to a broader population than previous studies. However, our exploratory approach using nominal p-value cutoffs may necessitate replication of these findings in another study. While novel application of dietary patterns summarizing candidate metabolites did not successfully extend outside of the nested case-control study, the approach may show promise for future work with targeted measurement of disease-related metabolites. Application of these methods that account for complex dietary intake are particularly important in type 1 diabetes research, since components of dietary intake are among the leading hypothesized environmental factors that act on high-risk genetic background to cause type 1 diabetes.

In the TEDDY study, higher levels of dicarboxylic acids and lower level PCs, SMs, PEs, EtherPLs, and GlcCers at 9-months of age was associated with increased risk of mAb+. Characterization of this profile may help shape the future of early diagnosis or prevention efforts.

## Supplementary information

Supplement

## Data Availability

Data described in the manuscript and code book will be made available upon request from the NIDDK Central Repository at https://www.niddkrepository.org/studies/teddy.
