# Supplementary material for: Metabolite-related dietary patterns and the development of islet autoimmunity
Source: Sci Rep. 2019 Oct 15;9:14819. doi: 10.1038/s41598-019-51251-4 (PMC6794249; doi:10.1038/s41598-019-51251-4)
Supplement: Supplementary file 1 — Supplement [file 41598_2019_51251_MOESM1_ESM.pdf]

Metabolite-related dietary patterns and the development of islet autoimmunity

Randi K Johnson[1], Lauren Vanderlinden[2], Brian C DeFelice[3], Katerina Kechris[2], Ulla Uusitalo[4], Oliver Fiehn[3,5], Marci Sontag[1], Tessa Crume[1], Andreas Beyerlein[6,7], Åke Lernmark[8], Jorma Toppari[9,10], Anette-G Ziegler[7], Jin-Xiong She[11], William Hagopian[12], Marian Rewers[13], Beena Akolkar[14], Jeffrey Krischer[4], Suvi M Virtanen[15,16,17], and Jill M Norris[1]*, for the TEDDY Study Group[†]

Supplemental Figure 1—Summary of metabolomics data pre-processing pipeline in all TEDDY metabolomics samples.

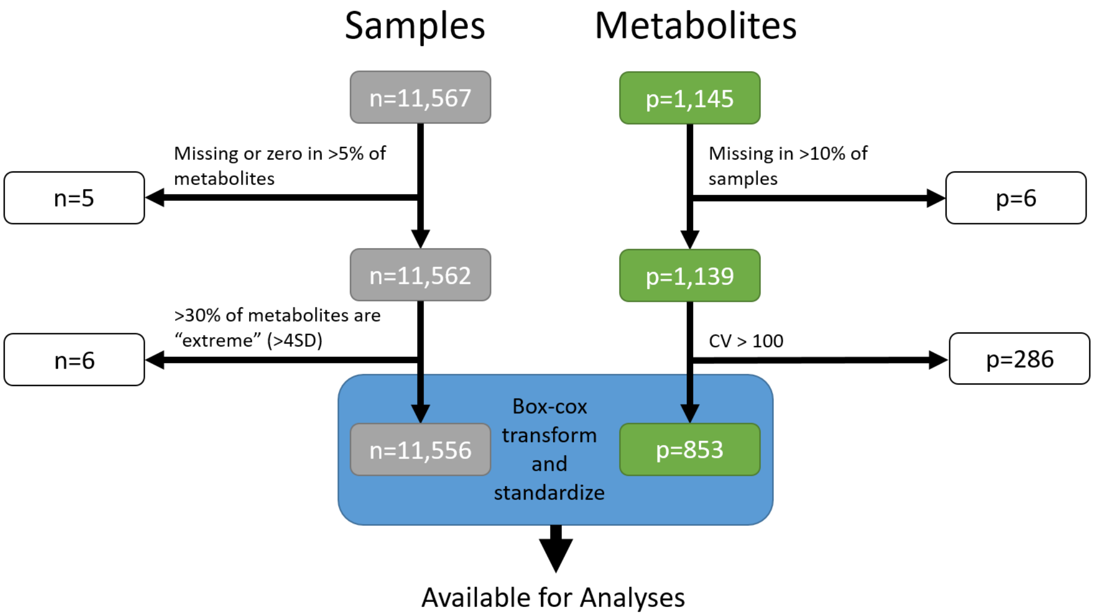

n=number of samples, p=number of metablites/features, CV=coefficient of variation, SD=standard deviation

Supplemental Table 1: Chemically identified metabolites used in ChemRICH metabolomics set enrichment analysis

Variable	Compound Name	InChiKeys	Pubchem ID	SMILES
t74	3-aminoisobutyric acid	QCHPKSFMDHPSNR-UHFFFAOYSA-N	25201103	CC(C[NH3+])C(=O)[O-]
t147	Acylcarnitine (C10:1)	GOOOCIIXFLVRAG-UHFFFAOYSA-N	53481651	C[N+](C)(C)CC(CC(=O)[O-])OC(=O)CCCCCCCC=C
t148	Acylcarnitine (C14:2)	HXOGMKPCIDSSKJ-NKBLVAAJSA-N	53481681	CCCCCCCC/C=C/C=C/CC(=O)O[C@@H](CCC(=O)[O-])[N+](C)(C)C
t149	Acylcarnitine (C18:3)	DFVGGGHKDAHYIU-UHMZJXMFSA-N	53477821	CC/C=C\C/C=C\C/C=C\CCCCCCCC(=O)O[C@@H](CCC(=O)[O-])[N+](C)(C)C
t150	Acylcarnitine (C8:0)	CXTATJFJDMJMIY-CYBMUJFWSA-N	11953814	CCCCCCCC(=O)O[C@H](CC(=O)[O-])C[N+](C)(C)C
t151	Acylcarnitine (C8:1)	YMIVWYONPRZBEJ-LXKVQUBZSA-N	53481667	CCCCC/C=C/C(=O)O[C@@H](CCC(=O)[O-])[N+](C)(C)C
t141	Acylcarnitine C10:0	LZOSYCMHQXPBFU-UHFFFAOYSA-N	10245190	CCCCCCCCCC(=O)OC(CC(=O)[O-])C[N+](C)(C)C
t142	Acylcarnitine C12:0	FUJLYHJROOYKRA-QGZVFWFLSA-N	168381	CCCCCCCCCCCC(=O)O[C@H](CC(=O)[O-])C[N+](C)(C)C
t144	Acylcarnitine C18:0	FNPHNLNTJNMAEE-UHFFFAOYSA-N	6426855	CCCCCCCCCCCCCCCCCC(=O)OC(CC(=O)[O-])C[N+](C)(C)C
t145	Acylcarnitine C18:1	HITOYGLMAFIRNI-YSESTWPTSA-N	53477830	CCCCCC/C=C/CCCCCCCCCC(=O)O[C@@H](CCC(=O)[O-])[N+](C)(C)C
t146	Acylcarnitine C18:2	MJLXQSQYKZWZCB-DQFWFXSYSA-N	6450015	CCCCC/C=C\C/C=C\CCCCCCCC(=O)O[C@H](CC(=O)[O-])C[N+](C)(C)C
t73	adipic acid	WNLRTRBMVRJNCN-UHFFFAOYSA-N	196	C(CCC(=O)O)CC(=O)O
t72	alanine	QNAYBMKLOCPYGJ-REOHCLBHSA-N	5950	C[C@@H](C(=O)O)N
t70	asparagine	DCXYFEDJOCDNAF-REOHCLBHSA-N	6267	C([C@@H](C(=O)O)N)C(=O)N
t69	aspartic acid	CKLJMWTZIZZHCS-REOHCLBHSA-N	44367445	C([C@@H](C(=O)O)[NH3+])C(=O)[O-]
t68	benzoic acid	WPYMKLBDIGXBTP-UHFFFAOYSA-N	20144841	[H+].C1=CC=C(C=C1)C(=O)[O-]
t67	capric acid	GHVNFZFCNZKVNT-UHFFFAOYSA-N	2969	CCCCCCCCCC(=O)O

t66	caprylic acid	WWZKQHOCKIZLMA-UHFFFAOYSA-N	379	CCCCCCCC(=O)O
t433	CE (16:1)	HODJWNWCVNUPAQ-FSAOOAOSSA-N	22833543	CCCCCC/C=C\CCCCCCCC(=O)OC1CC[C@@]2(C3CC[C@@]4([C@H](CCC4C3CC=C2C1)[C@H](C)CCCC(C)C)C)C
t434	CE (18:1)	RJECHNNFRHZQKU-RMUVNZEASA-N	5283632	CCCCCCCC/C=C\CCCCCCCC(=O)O[C@H]1CC[C@@]2([C@H]3CC[C@]4([C@H]([C@@H]3CC=C2C1)CC[C@@H]4[C@H](C)CCCC(C)C)C)C
t435	CE (18:2)	NAACPBBQTFFYQB-LJAITQKLSA-N	5287939	CCCCC/C=C\C/C=C\CCCCCCCC(=O)O[C@H]1CC[C@@]2([C@H]3CC[C@]4([C@H]([C@@H]3CC=C2C1)CC[C@@H]4[C@H](C)CCCC(C)C)C)C
t436	CE (18:3)	FYMCIBHUFSIWCE-WVXFKAQASA-N	6436907	CC/C=C\C/C=C\C/C=C\CCCCCCCC(=O)O[C@H]1CC[C@@]2([C@H]3CC[C@]4([C@H]([C@@H]3CC=C2C1)CC[C@@H]4[C@H](C)CCCC(C)C)C)C
t152	CE (20:3)	MLPRJPSMAFZPLA-PJSAOELNSA-N	53477892	CCCCC/C=C\C/C=C\C/C=C\CCCCCCC(=O)O[C@H]1CC[C@@]2(C3CC[C@]4(C(C3CC=C2C1)CCC4[C@H](C)CCCC(C)C)C)C
t437	CE (20:4)	IMXSFYNMSOULQS-BEDFLICRSA-N	6479222	CCCCC/C=C\C/C=C\C/C=C\C/C=C\CCCC(=O)O[C@H]1CC[C@@]2([C@H]3CC[C@]4([C@H]([C@@H]3CC=C2C1)CC[C@@H]4[C@H](C)CCCC(C)C)C)C
t438	CE (20:5)	XZFUGMCJZFRBKF-JIKDAPOUSA-N	53477889	CC/C=C\C/C=C\C/C=C\C/C=C\C/C=C\CCCC(=O)O[C@H]1CC[C@@]2(C3CC[C@]4(C(C3CC=C2C1)CCC4[C@H](C)CCCC(C)C)C)C
t439	CE (22:6)	VOEVEGPMRIYYKC-HNJOWPRISA-N	14274978	CC/C=C\C/C=C\C/C=C\C/C=C\C/C=C\C/C=C\CCC(=O)O[C@H]1CC[C@@]2([C@H]3CC[C@]4([C@H]([C@@H]3CC=C2C1)CC[C@@H]4[C@H](C)CCCC(C)C)C)C
t848	Ceramide (d33:1)	QBFXCLDNTKBAPQ-STSAHMJASA-N	52931112	CCCCCCCCCCCCCCCC(=O)N[C@@H](CO)[C@@H](/C=C/CCCCCCCCCCC)O
t849	Ceramide (d34:0)	GCGTXOVNNFGTPQ-JHOUSYSJSA-N	5283572	CCCCCCCCCCCCCCC[C@H]([C@H](CO)NC(=O)CCCCCCCCCCCCCCC)O
t850	Ceramide (d34:1)	YDNKGFDKKRUKPY-TURZORIXSA-N	5283564	CCCCCCCCCCCCCCCC(=O)N[C@@H](CO)[C@@H](/C=C/CCCCCCCCCCCCC)O
t851	Ceramide (d34:2)	XXWRZIYYFPIQHE-XPAOSYCESA-N	52931118	CCCCCCCCCCCCCCCC(=O)N[C@@H](CO)[C@@H](/C=C/CCCCCCCC/C=C\CCC)O
t852	Ceramide (d36:1)	MJQIARGPQMNBGT-WWUCIAQXSA-N	6442676	CCCCCCCCCCCCCCC[C@H]([C@H](CO)NC(=O)CCCCCCC/C=C\CCCCCCCC)O
t853	Ceramide (d38:1)	XWBWIAOWSABHFI-NUKVNZTCSA-N	5283566	CCCCCCCCCCCCCCCCCCCC(=O)N[C@@H](CO)[C@@H](/C=C/CCCCCCCCCCCCC)O
t854	Ceramide (d39:1)	WYSRACVJQVNCRW-PQPBPFPMSA-N	11273482	CCCCCCCCCCCCCCCCCCCCCCC(=O)N[C@@H](CO)[C@@H](/C=C/CCCCCCCCCCC)O
t855	Ceramide (d40:0)	SXPRAKSDHOEHIG-ZESVVUHVSA-N	5283575	CCCCCCCCCCCCCCCCCCCCCCC(=O)N[C@@H](CO)[C@@H](CCCCCCCCCCCCCCC)O
t856	Ceramide (d40:1)	KEPQASGDXIEOIL-GLQCRSEXSA-N	5283567	CCCCCCCCCCCCCCCCCCCCCC(=O)N[C@@H](CO)[C@@H](/C=C/CCCCCCCCCCCCC)O

t857	Ceramide (d40:2)	HILTUFAERVOALR-MQXYEJFFSA-N	52931123	CCCCCCCCCCCCCCCCCCCCC(=O)N[C@@H](CO)[C@@H](/C=C/CCCCCCC/C=C\CCC)O
t859	Ceramide (d42:0)	BPLYVSYSBPLDOA-WVILEFPPSA-N	5283577	CCCCCCCCCCCCCCCCCCCCCCCC(=O)N[C@@H](CO)[C@@H](CCCCCCCCCCCCCCC)O
t860	Ceramide (d42:1)	ZJVVOYPTFQEGPH-AUTSUKAISA-N	5283571	CCCCCCCCCCCCCCCCCCCCCCC(=O)N[C@@H](CO)[C@@H](/C=C/CCCCCCCCCCCCC)O
t861	Ceramide (d42:2)	VJSBNBBOSZJDKB-KPEYJIHVSA-N	5283568	CCCCCCCCCCCCC/C=C/[C@H]([C@H](CO)NC(=O)CCCCCCCCCCCCC/C=C\CCCCCCCC)O
t863	Ceramide (d43:1)	QHPYSHVSWAOLHS-PVNBSDFKSA-N	9547202	CCCCCCCCCCCCCCCCCCCCCCCCC(=O)N[C@@H](CO)[C@@H](/C=C/CCCCCCCCCCCCC)O
t864	Ceramide (d44:1)	CJROVRTUSFQVMR-GVOPMEMSSA-N	5283570	CCCCCCCCCCCCCCCCCCCCCCCCC(=O)N[C@@H](CO)[C@@H](/C=C/CCCCCCCCCCCCC)O
t64	citramalic acid	XFTRTWQBIOMVPK-UHFFFAOYSA-N	1081	CC(CC(=O)O)(C(=O)O)O
t63	creatinine	DDRJAANPRJIHGJ-UHFFFAOYSA-N	588	CN1CC(=O)N=C1N
t62	cystine	LEVWYRKDKASIDU-UHFFFAOYSA-N	24798687	C(C(C(=O)[O-])[NH3+])SSCC(C(=O)[O-])[NH3+]
t61	deoxypentitol	FJGNTEKSQVNVTJ-UHFFFAOYSA-N	270738	CC(C(C(CO)O)O)O
t444	DG (32:1)	XEQQGHISHUGMIP-ASUORMEESA-N	14275341	CCCCCCCCCCCCCC(=O)OC[C@H](CO)OC(=O)CCCCCCC/C=C\CCCCCCC
t445	DG (34:1)	YEJYLHKQOBOSCP-OZKTZCCCSA-N	5282283	CCCCCCCCCCCCCCC(=O)OC[C@H](CO)OC(=O)CCCCCCC/C=C\CCCCCCC
t156	DG (36:1)	SAEPUUXWQQNLGN-LVVMQYBKSA-N	6443547	CCCCCCCCCCCCCCCCC(=O)OC[C@H](CO)OC(=O)CCCCCCC/C=C\CCCCCCCC
t447	DG (36:2)	AFSHUZFNMVJNKX-LLWMBOQKSA-N	9543716	CCCCCCCC/C=C\CCCCCCCC(=O)OC[C@H](CO)OC(=O)CCCCCCC/C=C\CCCCCCCC
t448	DG (36:3)	BLZVZPYMHLXLHG-JOBMVARSSA-N	9543722	CCCCCCCC/C=C\CCCCCCCC(=O)OC[C@H](CO)OC(=O)CCCCCCC/C=C\C/C=C\CCCCC
t157	DG (36:4)	MQGBAQLIFKSMEM-ZHARMHCNSA-N	9543729	CCCCC/C=C\C/C=C\CCCCCCCC(=O)OC[C@H](CO)OC(=O)CCCCCCC/C=C\C/C=C\CCCCC
t159	DG (36:5)	PGXBELQFNRPKBC-WBVIKXMWSA-N	9543737	CCCCC/C=C\C/C=C\CCCCCCCC(=O)OC[C@H](CO)OC(=O)CCCCCCC/C=C\C/C=C\C/C=C\CC
t160	DG (38:0)	IQNYOCFHHRCMKY-KDXMTYKHSA-N	53478362	CCCCCCCCCCCCCCCCCCCCC(=O)OC[C@H](CO)OC(=O)CCCCCCCCCCCCCCC
t161	DG (38:3)	ADXAWIIUCSQOAS-SMJOZILHSA-N	9543766	CCCCCCCC/C=C\CCCCCCCC(=O)OC[C@H](CO)OC(=O)CCCCCCCCC/C=C\C/C=C\CCCCC

t449	DG (38:5)	GRGDLDNREYVILP-CNWVQWJYSA-N	9543784	CCCCC/C=C\C/C=C\CCCCCCCCCC(=O)O[C@@H](CO)COC(=O)CCCCCCC/C=C\C/C=C\C/C=C\CC
t450	DG (38:6)	YDVDXUYJFQLPEG-CTHJWPIASA-N	9543795	CCCCC/C=C\C/C=C\C/C=C\CCCCCCC(=O)O[C@@H](CO)COC(=O)CCCCCCC/C=C\C/C=C\C/C=C\CC
t60	erythronic acid lactone	SGMJBNSHAZVGMC-PWNYCUMCSA-N	5325915	C1[C@H]([C@H](C(=O)O1)O)O
t514	FA (16:0)	IPCSVZSSVZVIGE-UHFFFAOYSA-N	985	CCCCCCCCCCCCCCCC(=O)O
t515	FA (16:1)	SECPZKHBENQXJG-FPLPWBNLSA-N	445638	CCCCCC/C=C\CCCCCCCC(=O)O
t516	FA (18:0)	QIQXTHQIDYTFRH-UHFFFAOYSA-N	5281	CCCCCCCCCCCCCCCCCC(=O)O
t517	FA (18:1)	ZQPPMHVWECSIRJ-KTKRTIGZSA-N	445639	CCCCCCCC/C=C\CCCCCCCC(=O)O
t518	FA (18:2)	JBYXPOFIGCOSSB-XBLVEGMJSA-N	5282796	CCCCCC/C=C/C=C/CCCCCCCC(=O)O
t519	FA (20:4)	YZXBAPSDXZZRGB-DOFZRALJSA-N	444899	CCCCC/C=C\C/C=C\C/C=C\C/C=C\CCCC(=O)O
t520	FA (22:4)	TWSWSIQAPQLDBP-DOFZRALJSA-N	5497181	CCCCC/C=C\C/C=C\C/C=C\C/C=C\CCCCCC(=O)O
t59	fucose	SHZGCJCMOBCMKK-FPRJBGLDSA-N	439650	C[C@@H]1[C@@H]([C@@H]([C@H]([C@@H](O1)O)O)O)O
t58	galactonic acid	RGHNJXZEOKUKBD-MGCNEYSASA-N	128869	C([C@H]([C@@H]([C@@H]([C@H](C(=O)O)O)O)O)O)O
t451	Gal-Gal-Cer(d18:1/16:0) or Lactosylceramide(d18:1/16:0)	HLIJNIKSBCIDGO-QKLMXXKVSA-N	53477895	CCCCCCCCCCCCCCCC(=O)N[C@@H](CO[C@H]1[C@@H]([C@H]([C@@H]([C@H](O1)CO)O[C@H]2[C@@H]([C@H]([C@H]([C@H](O2)CO)O)O)O)O)O)[C@@H](/C=C/CCCCCCCCCCCCC)O
t865	GlcCer (d38:1)	DFELABABMXOKTD-IYFIADHGSA-N	20057356	CCCCCCCCCCCCCCCCCCCC(=O)N[C@@H](CO[C@H]1[C@@H]([C@H]([C@@H]([C@H](O1)CO)O)O)O)[C@@H](/C=C/CCCCCCCCCCCCC)O
t452	GlcCer (d40:1)	QMYGQSYNQBLKGY-YAEABVQUSA-N	70699232	CCCCCCCCCCCCCCCCCCCCCCCC(=O)N[C@@H](CO[C@H]1C(C([C@@H]([C@H](O1)CO)O)O)O)[C@@H](/C=C/CCCCCCCCCCC)O
t867	GlcCer (d41:1)	SJGWLQDELUWRDA-MUYAOIFFSA-N	52931253	CCCCCCCCCCCCCCCCCCCCCC(=O)N[C@@H](CO[C@H]1C(C([C@@H]([C@H](O1)CO)O)O)O)[C@@H](/C=C/CCCCCCCCCCCCC)O
t453	GlcCer (d42:1)	SNVYJLGKALMFOP-MOLROICHSA-N	6321367	CCCCCCCCCCCCCCC[C@H]([C@H](CO[C@H]1C(C([C@@H]([C@H](O1)CO)O)O)O)NC(=O)CCCCCCCCCCCCC/C=C\CCCCCCCC)O
t454	GlcCer (d42:2)	WBOZIXHPUPAOIA-RBELZSLISA-N	6321360	CCCCCCCCCCCC/C=C/[C@H]([C@H](CO[C@H]1C(C([C@@H]([C@H](O1)CO)O)O)O)NC(=O)CCCCCCCCCCCCC/C=C\CCCCCCCC)O

t870	GlcCer(d14:1(4E)/20:0(2OH))	YYILQTLJZBSOCA-IMJQGFQJSA-N	70699246	CCCCCCCCCCCCCCCCCC(C(=O)N[C@@H](CO[C@H]1C(C([C@@H]([C@H](O1)CO)O)O)O)[C@@H](/C=C/CCCCCCCCC)O)O
t57	gluconic acid	RGHNJXZEOKUKBD-QTBDOELSSA-N	6857417	C([C@@H]([C@H]([C@@H]([C@@H](C(=O)O)O)O)O)O)O
t56	glucose	WQZGKKKJIJFFOK-VFUOTHLCSA-N	64689	C([C@@H]1[C@H]([C@@H]([C@H]([C@@H](O1)O)O)O)O)O
t55	glutamine	ZDXPYRJPNDTMRX-VKHMYHEASA-N	5961	C(CC(=O)N)[C@@H](C(=O)O)N
t54	glutaric acid	JFCQEDHGNNZCLN-UHFFFAOYSA-N	23322899	[H+].[H+].C(CC(=O)[O-])CC(=O)[O-]
t53	glycerol	PEDCQBHIVMGVHV-UHFFFAOYSA-N	753	C(C(CO)O)O
t52	glycine	DHMQDGOQFOQNFH-UHFFFAOYSA-N	5257127	C(C(=O)[O-])[NH3+]
t51	heptadecanoic acid	KEMQGTRYUADPNZ-UHFFFAOYSA-N	10465	CCCCCCCCCCCCCCCCC(=O)O
t50	hexitol	FBPFZTCFMRRESA-UHFFFAOYSA-N	453	C(C(C(C(C(CO)O)O)O)O)O
t49	histidine	HNDVDQJCIGZPNO-YFKPBYRVSA-N	6274	C1=C(NC=N1)C[C@@H](C(=O)O)N
t47	hydroxylamine	AVXURJPOCDRRFD-UHFFFAOYSA-N	787	NO
t46	indole-3-acetate	SEOVTRFCIGRIMH-UHFFFAOYSA-N	802	C1=CC=C2C(=C1)C(=CN2)CC(=O)O
t45	indoxyl sulfate	BXFFHSIDQOFMLE-UHFFFAOYSA-N	10258	C1=CC=C2C(=C1)C(=CN2)OS(=O)(=O)O
t44	isoleucine	AGPKZVBTJJNPAG-WHFBIAKZSA-N	6306	CC[C@H](C)[C@@H](C(=O)O)N
t43	isothreonic acid	JPIJQSOTBSSVTP-GBXIJSLDSA-N	151152	C([C@H]([C@@H](C(=O)O)O)O)O
t42	lactic acid	JVTAAEKCZFNVCJ-UHFFFAOYSA-N	19789253	[H+].CC(C(=O)[O-])O
t455	Lactosylceramide (d18:1/24:1(15Z))	MKOKWBRPIBQYJJ-LWQSSKHKSA-N	20057309	CCCCCCCCCCCCC/C=C/[C@H]([C@H](CO[C@H]1C([C@H]([C@@H]([C@H](O1)CO)O[C@H]2[C@@H]([C@H]([C@H]([C@H](O2)CO)O)O)O)O)O)NC(=O)CCCCCCCCCCCCC/C=C\CCCCCCCC)O
t41	lauric acid	POULHZVOKOAJMA-UHFFFAOYSA-N	3893	CCCCCCCCCCCC(=O)O
t40	leucine	ROHFNLRQFUQHCH-YFKPBYRVSA-N	6106	CC(C)C[C@@H](C(=O)O)N

t39	linoleic acid	OYHQOLUKZRVURQ-HZJYTTRNSA-N	5280450	CCCCC/C=C\C/C=C\CCCCCCCC(=O)O
t521	LPC (14:0)	VXUOFDJKYGDUJI-OAQYLSRUSA-N	460604	CCCCCCCCCCCCCC(=O)OC[C@H](COP(=O)([O-])OCC[N+](C)(C)C)O
t163	LPC (15:0)	RJZVWDTYEWCUAR-JOCHJYFZSA-N	24779458	CCCCCCCCCCCCCCC(=O)OC[C@H](COP(=O)([O-])OCC[N+](C)(C)C)O
t871	LPC (16:0)	ASWBNKHCZGQVJV-HSZRJFAPSA-N	460602	CCCCCCCCCCCCCCCC(=O)OC[C@H](COP(=O)([O-])OCC[N+](C)(C)C)O
t522	LPC (16:1)	LFUDDCMNKWEORN-ZXEGGCGDSA-N	24779461	CCCCCC/C=C\CCCCCCCC(=O)OC[C@H](COP(=O)([O-])OCC[N+](C)(C)C)O
t166	LPC (17:1)	LPMGFNAQZPADDZ-FJIRUFBNSA-N	24779451	CCCCCCC/C=C\CCCCCCCC(=O)OC[C@H](COP(=O)([O-])OCC[N+](C)(C)C)O
t523	LPC (18:0)	IHNKQIMGVNPMTC-RUZDIDTESA-N	497299	CCCCCCCCCCCCCCCCCC(=O)OC[C@H](COP(=O)([O-])OCC[N+](C)(C)C)O
t525	LPC (18:1)	YAMUFBLWGFFICM-PTGWMXDISA-N	16081932	CCCCCCCC/C=C\CCCCCCCC(=O)OC[C@H](COP(=O)([O-])OCC[N+](C)(C)C)O
t872	LPC (18:2)	SPJFYYJXNPEZDW-FTJOPAKQSA-N	11005824	CCCCC/C=C\C/C=C\CCCCCCCC(=O)OC[C@H](COP(=O)([O-])OCC[N+](C)(C)C)O
t170	LPC (18:3)	WKQNRCYKYCKESD-YVHLTTHBSA-N	24779469	CC/C=C\C/C=C\C/C=C\CCCCCCCC(=O)OC[C@H](COP(=O)([O-])OCC[N+](C)(C)C)O
t171	LPC (20:0)	UATOAILWGVYRQS-HHHXNRCGSA-N	24779473	CCCCCCCCCCCCCCCCCCCC(=O)OC[C@H](COP(=O)([O-])OCC[N+](C)(C)C)O
t526	LPC (20:1)	GJTDRNFWIDPARY-GTPZACKGSA-N	24779475	CCCCCCCCCC/C=C\CCCCCCCC(=O)OC[C@H](COP(=O)([O-])OCC[N+](C)(C)C)O
t527	LPC (20:2)	YYQVCMMXPIJVHY-ZOIJLGJPSA-N	52924053	CCCCC/C=C\C/C=C\CCCCCCCCCC(=O)OC[C@H](COP(=O)([O-])OCC[N+](C)(C)C)O
t528	LPC (20:3)	BBNHCUBQEQJHIG-FZZJNMCHSA-N	52924055	CCCCC/C=C\C/C=C\C/C=C\CCCCCCC(=O)OC[C@H](COP(=O)([O-])OCC[N+](C)(C)C)O
t175	LPC (20:4)	GOMVPVRDBLLHQC-VEJNOCSESA-N	53480469	CC/C=C\C/C=C\C/C=C\C/C=C\CCCCCCC(=O)OC[C@H](COP(=O)([O-])OCC[N+](C)(C)C)O
t176	LPC (20:5)	PDIGSOAOQOXRDU-WJPZTBRDSA-N	11757087	CC/C=C\C/C=C\C/C=C\C/C=C\C/C=C\CCCC(=O)OC[C@H](COP(=O)([O-])OCC[N+](C)(C)C)O
t177	LPC (22:4)	ZOJBSSVHFSBHMP-JJJSWPRASA-N	52924039	CCCCC/C=C\C/C=C\C/C=C\C/C=C\CCCCCC(=O)OC[C@H](COP(=O)([O-])OCC[N+](C)(C)C)O
t529	LPC (22:5)	YBUXFQUGNPBZPS-YNBHEIDWSA-N	53480473	CCCCC/C=C\C/C=C\C/C=C\C/C=C\C/C=C\CCC(=O)OC[C@H](COP(=O)([O-])OCC[N+](C)(C)C)O
t179	LPC (22:6)	LSOWKZULVQWMLY-APPDJCNMSA-N	10415542	CC/C=C\C/C=C\C/C=C\C/C=C\C/C=C\C/C=C\CCC(=O)OC[C@H](COP(=O)([O-])OCC[N+](C)(C)C)O

t180	LPC (o-16:0)	VLBPIWYTPAXCFJ-DEOSSOPVSA-N	10480367	CCCCCCCCCCCCCCCCOC[C@@H](COP(=O)([O-])OCC[N+](C)(C)C)O
t181	LPC (p-16:0) or LPC (o-16:1)	HTZINLFNXLXRBC-CQLBIITFSA-N	10917802	CCCCCCCCCCCCCC/C=C\OC[C@H](COP(=O)([O-])OCC[N+](C)(C)C)O
t530	LPE (16:0)	CKPBBEOJHAPPBT-HXUWFJFHSA-N	53480922	CCCCCCCCCCCCCCCC(=O)O[C@H](CO)COP(=O)(O)OCCN
t531	LPE (18:2)	DBHKHNGBVGWQJE-USWSLJGRSA-N	52925130	CCCCC/C=C\C/C=C\CCCCCCCC(=O)OC[C@H](COP(=O)(O)OCCN)O
t532	LPE (20:4)	JPNPIRVRGLGTRE-YSKCIPFOSA-N	53480952	CC/C=C\C/C=C\C/C=C\C/C=C\CCCCCCC(=O)OC[C@H](COP(=O)(O)OCCN)O
t533	LPE (22:6)	XEVRBOQZSXWGQO-PAUXXPOVSA-N	52925132	CC/C=C\C/C=C\C/C=C\C/C=C\C/C=C\C/C=C\CCC(=O)OC[C@H](COP(=O)(O)OCCN)O
t38	lysine	KDXKERNSBIXSRK-YFKPBYRVSA-N	5962	C(CCN)C[C@@H](C(=O)O)N
t37	lyxitol	HEBKCHPVOIAQTA-IMJSIDKUSA-N	439255	C([C@@H](C([C@H](CO)O)O)O)O
t36	malic acid	BJEPYKJPYRNKOW-UHFFFAOYSA-N	20130941	[H+].[H+].C(C(C(=O)[O-])O)C(=O)[O-]
t35	methanolphosphate	CAAULPUQFIIOTL-UHFFFAOYSA-N	13130	COP(=O)(O)O
t34	methionine	FFEARJCKVFRZRR-BYPYZUCNSA-N	6137	CSCC[C@@H](C(=O)O)N
t32	myo-inositol	CDAISMWEOUEBRE-UHFFFAOYSA-N	892	C1(C(C(C(C(C1O)O)O)O)O)O
t31	N-methylalanine	GDFAOVXKHJXLEI-VKHMYHEASA-N	5288725	C[C@@H](C(=O)O)NC
t30	nornicotine	MYKUKUCHPMASKF-UHFFFAOYSA-N	412	C1CC(NC1)C2=CN=CC=C2
t28	ornithine	AHLPHDHHMVZTML-BYPYZUCNSA-N	6262	C(C[C@@H](C(=O)O)N)CN
t27	oxoproline	ODHCTXKNWHHXJC-VKHMYHEASA-N	7405	C1CC(=O)N[C@@H]1C(=O)O
t184	PC (16:0/9:0(CHO))	PPTNNIINSOQWCE-WJOKGBTCSA-N	46907874	CCCCCCCCCCCCCCCC(=O)OC[C@H](COP(=O)([O-])OCC[N+](C)(C)C)OC(=O)CCCCCCCC=O
t185	PC (28:0)	CITHEXJVPOWHKC-UUWRZZSWSA-N	5459377	CCCCCCCCCCCCCC(=O)OC[C@H](COP(=O)([O-])OCC[N+](C)(C)C)OC(=O)CCCCCCCCCCCCC
t186	PC (30:0)	RFVFQQWKPSOBED-PSXMRANNSA-N	129657	CCCCCCCCCCCCCCCC(=O)O[C@H](COC(=O)CCCCCCCCCCCC)COP(=O)([O-])OCC[N+](C)(C)C

t187	PC (30:1)	ANKCYRKQDLQXGL-MRDDHZETSA-N	52922250	CCCCCCCCCCCCCCCC(=O)O[C@H](COC(=O)CCCCCCC/C=C\CCCC)COP(=O)([O-])OCC[N+](C)(C)C
t188	PC (31:0)	NPGWXTIWUUFYAB-DIPNUNPCSA-N	24778680	CCCCCCCCCCCCCCCC(=O)OC[C@H](COP(=O)([O-])OCC[N+](C)(C)C)OC(=O)CCCCCCCCCCCCCC
t189	PC (31:1)	QFVHCMLUKNHDSH-WTWBAFHPSA-N	24778657	CCCCCCCCCCCCCCC(=O)OC[C@H](COP(=O)([O-])OCC[N+](C)(C)C)OC(=O)CCCCCCC/C=C\CCCCCC
t873	PC (32:0)	KILNVBDSWZSGLL-KXQOOQHDSA-N	452110	CCCCCCCCCCCCCCCC(=O)OC[C@H](COP(=O)([O-])OCC[N+](C)(C)C)OC(=O)CCCCCCCCCCCCCCC
t534	PC (32:1)	QIBZFHLFHCIUOT-NPBIGWJUSA-N	6443788	CCCCCCCCCCCCCCCC(=O)OC[C@H](COP(=O)([O-])OCC[N+](C)(C)C)OC(=O)CCCCCCC/C=C\CCCCCC
t192	PC (32:2)	GPWHCUUIQMGELX-VHQDNGOZSA-N	24778764	CCCCCC/C=C\CCCCCCCC(=O)OC[C@H](COP(=O)([O-])OCC[N+](C)(C)C)OC(=O)CCCCCCC/C=C\CCCCCC
t193	PC (32:3)	UXEFXNOSLOCOLX-ZCHSEWAGSA-N	52922763	CCCCCCCCCCCCCC(=O)O[C@H](COC(=O)CCCC/C=C\C/C=C\C/C=C\CCCCC)COP(=O)([O-])OCC[N+](C)(C)C
t194	PC (33:0)	FHENRYRLCPXONH-LDLOPFEMSA-N	52922645	CCCCCCCCCCCCCCCCCC(=O)OC[C@H](COP(=O)([O-])OCC[N+](C)(C)C)OC(=O)CCCCCCCCCCCCCC
t196	PC (33:2)	SBNDHGBVMZMSNL-UESLNCBNSA-N	52922715	CCCCCCCCCCCCCCC(=O)O[C@H](COC(=O)CCCCCCC/C=C\C/C=C\CCCCC)COP(=O)([O-])OCC[N+](C)(C)C
t197	PC (34:0)	PZNPLUBHRSSFHT-RRHRGVEJSA-N	24778686	CCCCCCCCCCCCCCCCCC(=O)O[C@H](COC(=O)CCCCCCCCCCCCCCC)COP(=O)([O-])OCC[N+](C)(C)C
t874	PC (34:1)	WTJKGGKOPKCXLL-VYOBOKEXSA-N	5497103	CCCCCCCCCCCCCCCC(=O)OC[C@H](COP(=O)([O-])OCC[N+](C)(C)C)OC(=O)CCCCCCC/C=C\CCCCCCCC
t875	PC (34:2)	JLPULHDHAOZNQI-ZTIMHPMXSA-N	5287971	CCCCCCCCCCCCCCCC(=O)OC[C@H](COP(=O)([O-])OCC[N+](C)(C)C)OC(=O)CCCCCCC/C=C\C/C=C\CCCCC
t539	PC (34:3)	CNNSEHUKQJCGTE-UPPWDXJYSA-N	24778699	CCCCCCCCCCCCCCCC(=O)OC[C@H](COP(=O)([O-])OCC[N+](C)(C)C)OC(=O)CCCC/C=C\C/C=C\C/C=C\CCCCC
t200	PC (34:4)	YWDDIWXKFJEMKF-JTZVLWBESA-N	52922891	CCCCCCCCCCCCCCCC(=O)O[C@H](COC(=O)CCCC/C=C\C/C=C\C/C=C\C/C=C\CC)COP(=O)([O-])OCC[N+](C)(C)C
t201	PC (35:1)	MFHIZGSSDZJFKD-IYEJTHTFSA-N	52922679	CCCCCCCCCCCCCCCCC(=O)O[C@H](COC(=O)CCCCCCC/C=C\CCCCCCCC)COP(=O)([O-])OCC[N+](C)(C)C
t541	PC (35:2)	ZSKWZJYUVZYDQU-WESJWMGVSA-N	52922491	CCCCCCCCCCCCCCCCC(=O)OC[C@H](COP(=O)([O-])OCC[N+](C)(C)C)OC(=O)CCCCCCC/C=C\C/C=C\CCCCC
t203	PC (35:2) B	LNGBVAOHJZCRIL-GPDPEMMZSA-N	52923157	CCCCCCCCCCCCCCC(=O)O[C@H](COC(=O)CCCCCCCCC/C=C\C/C=C\CCCCC)COP(=O)([O-])OCC[N+](C)(C)C
t204	PC (35:3)	AYXGHIQPMDYMJC-AHMBLZLYSA-N	52924614	CCCCCCCCCCCCCCCCCC(=O)O[C@H](COC(=O)CCCCCC/C=C\C/C=C\C/C=C\CCCCC)COP(=O)(O)OCCN
t205	PC (35:4)	OROZWUJCDDCYAU-IPUAOQJZSA-N	52922204	CCCCCCCCCCCCC(=O)OC[C@H](COP(=O)([O-])OCC[N+](C)(C)C)OC(=O)CCCCC/C=C\C/C=C\C/C=C\C/C=C\CCCCC

t543	PC (36:1)	ATHVAWFAEPLPPQ-VRDBWYNSSA-N	24778825	CCCCCCCCCCCCCCCCCC(=O)OC[C@H](COP(=O)([O-])OCC[N+](C)(C)C)OC(=O)CCCCCCC/C=C\CCCCCCCC
t876	PC (36:2)	SNKAWJBJQDLSFF-NVKMUCNASA-N	10350317	CCCCCCCC/C=C\CCCCCCCC(=O)OC[C@H](COP(=O)([O-])OCC[N+](C)(C)C)OC(=O)CCCCCCC/C=C\CCCCCCCC
t545	PC (36:3)	YPAZQMWFRMHBBM-CLKMJQEKSA-N	53478785	CCCCCC/C=C\CCCCCCCCCC(=O)O[C@H](COC(=O)CCCCCCC/C=C\C/C=C\CCCCC)COP(=O)([O-])OCC[N+](C)(C)C
t208	PC (36:3) A	BXRLDROZWDUSGM-ZRYFCQOPSA-N	24778937	CCCCCCCC/C=C\CCCCCCCC(=O)OC[C@H](COP(=O)([O-])OCC[N+](C)(C)C)OC(=O)CCCC/C=C\C/C=C\CCCCCCCC
t211	PC (36:4) A	NKQPOVROGSWLTO-NVPMBMBWSA-N	52922783	CCCCCCCC/C=C\CCCCCCCC(=O)O[C@H](COC(=O)CCCC/C=C\C/C=C\C/C=C\CCCCC)COP(=O)([O-])OCC[N+](C)(C)C
t212	PC (36:4) C	IIZPXYDJLKNOIY-JXPKJXOSSA-N	10747814	CCCCCCCCCCCCCCCC(=O)OC[C@H](COP(=O)([O-])OCC[N+](C)(C)C)OC(=O)CCC/C=C\C/C=C\C/C=C\C/C=C\CCCCC
t548	PC (36:5)	DYDDZDMJSQYFGN-OIVUZXIWSA-N	24778771	CCCCCC/C=C\CCCCCCCC(=O)OC[C@H](COP(=O)([O-])OCC[N+](C)(C)C)OC(=O)CCC/C=C\C/C=C\C/C=C\C/C=C\CCCCC
t214	PC (36:5) B	SUZYROYNFNQALJ-MHEIZRSESA-N	52923341	CCCCCCCCCCCCCCCC(=O)O[C@H](COC(=O)CCC/C=C\C/C=C\C/C=C\C/C=C\C/C=C\CC)COP(=O)([O-])OCC[N+](C)(C)C
t215	PC (36:6)	SPWBDEZMKCRQSX-NGPPOSSDSA-N	52922847	CCCCC/C=C\C/C=C\C/C=C\CCCCC(=O)O[C@H](COC(=O)CCCCCCC/C=C\C/C=C\C/C=C\CC)COP(=O)([O-])OCC[N+](C)(C)C
t550	PC (37:2)	MCZUABDVGPPWPM-HJTCUGKVSA-N	52922735	CCCCCCCCCCCCCCCCCCC(=O)O[C@H](COC(=O)CCCCCCC/C=C\C/C=C\CCCCC)COP(=O)([O-])OCC[N+](C)(C)C
t217	PC (37:3)	OOYQEEUUQRMQKL-JUUDQZDJSA-N	52922851	CCCCCCCCCCCCCCCCCCC(=O)O[C@H](COC(=O)CCCCCCC/C=C\C/C=C\C/C=C\CC)COP(=O)([O-])OCC[N+](C)(C)C
t218	PC (37:4)	QRPUCJXFPYFTMB-FBFLODOBSA-N	52922853	CCCCCCCCC/C=C\CCCCCCCC(=O)O[C@H](COC(=O)CCCCCCC/C=C\C/C=C\C/C=C\CC)COP(=O)([O-])OCC[N+](C)(C)C
t219	PC (37:5)	URYYGMVXBWUJFP-LQYSTYLLSA-N	53478655	CCCCCCCCCCCCCCC(=O)OC[C@H](COP(=O)([O-])OCC[N+](C)(C)C)OC(=O)CCCCC/C=C\C/C=C\C/C=C\C/C=C\C/C=C\CC
t220	PC (37:6)	GEINPYKZLFHHIL-HEXXMCQTSA-N	52922342	CCCCCCCCCCCCCCC(=O)OC[C@H](COP(=O)([O-])OCC[N+](C)(C)C)OC(=O)CC/C=C\C/C=C\C/C=C\C/C=C\C/C=C\C/C=C\CC
t552	PC (38:2)	KXXLFCAPKGRXBT-FMJYHZMHSA-N	24779263	CCCCCCCCCCCCCCCCCCCCC(=O)OC[C@H](COP(=O)([O-])OCC[N+](C)(C)C)OC(=O)CCCCCCC/C=C\C/C=C\CCCCC
t877	PC (38:3)	OJHJKEBRZSDTTL-VHWCKNCUSA-N	52922741	CCCCCCCC/C=C\CCCCCCCCCC(=O)O[C@H](COC(=O)CCCCCCC/C=C\C/C=C\CCCCC)COP(=O)([O-])OCC[N+](C)(C)C
t223	PC (38:4) A	PSVRFUPOQYJOOZ-QNPWAGBNSA-N	16219824	CCCCCCCCCCCCCCCCCC(=O)OC[C@H](COP(=O)([O-])OCC[N+](C)(C)C)OC(=O)CCC/C=C\C/C=C\C/C=C\C/C=C\CCCCC
t224	PC (38:4) B	DNYKSJQVBCVGOF-LCKGXUDJSA-N	52923291	CCCCCCCCCCCCCCCCCC(=O)O[C@H](COC(=O)CCC/C=C\C/C=C\C/C=C\C/C=C\CCCCC)COP(=O)([O-])OCC[N+](C)(C)C
t553	PC (38:5)	YLWBKBDNHWQEFU-YJXJLLHLSA-N	53479033	CCCCCC/C=C\CCCCCCCCCC(=O)O[C@H](COC(=O)CCC/C=C\C/C=C\C/C=C\C/C=C\CCCCC)COP(=O)([O-])OCC[N+](C)(C)C

t225	PC (38:5) A	SUACBSWYGWBPFC-GPUJSUHJSA-N	52923235	CCCCC/C=C\C/C=C\CCCCCCCC(=O)O[C@H](COC(=O)CCCCCC/C=C\C/C=C\C/C=C\CCCCC)COP(=O)([O-])OCC[N+](C)(C)C
t555	PC (38:6)	PLZBTDKJYHXIEW-DZUXOTHRSA-N	52923295	CCCCC/C=C\C/C=C\CCCCCCCC(=O)O[C@H](COC(=O)CCC/C=C\C/C=C\C/C=C\C/C=C\CCCCC)COP(=O)([O-])OCC[N+](C)(C)C
t229	PC (38:7)	BNNUJTATKJXKJP-XCDHYEIISA-N	53479075	CCCCC/C=C\C/C=C\C/C=C\CCCCC(=O)O[C@H](COC(=O)CCCCCC/C=C\C/C=C\C/C=C\C/C=C\CC)COP(=O)([O-])OCC[N+](C)(C)C
t230	PC (39:6)	QMCWOGICYCFNBF-BWHZRABLSA-N	52922637	CCCCC/C=C\C/C=C\C/C=C\C/C=C\CCCCCC(=O)O[C@H](COC(=O)CCCCCCC/C=C\C/C=C\CCCC)COP(=O)([O-])OCC[N+](C)(C)C
t556	PC (40:4)	QQIYXJBHMDYXHH-NMUBDWGHSA-N	52923573	CCCCC/C=C\C/C=C\CCCCCCCCCCCC(=O)OC[C@H](COP(=O)([O-])OCC[N+](C)(C)C)OC(=O)CCCCCCC/C=C\C/C=C\CCCCC
t558	PC (40:5)	LJFKFKIYUJNFPZ-ZLFSCUDPSA-N	52923133	CCCCCCCC/C=C\CCCCCCCCCC(=O)OC[C@H](COP(=O)([O-])OCC[N+](C)(C)C)OC(=O)CCC/C=C\C/C=C\C/C=C\C/C=C\CCCCC
t232	PC (40:5) A	IJTJDJOOHZVSAC-NDRUHXFFSA-N	53479083	CCCCCCCC/C=C\CCCCCCCCCC(=O)O[C@H](COC(=O)CCCCCC/C=C\C/C=C\C/C=C\C/C=C\CC)COP(=O)([O-])OCC[N+](C)(C)C
t233	PC (40:5) B	SFESOYFQZQJCOY-FXYWPAEZSA-N	52923365	CCCCCCCCCCCCCCCCCCCC(=O)O[C@H](COC(=O)CCC/C=C\C/C=C\C/C=C\C/C=C\C/C=C\CC)COP(=O)([O-])OCC[N+](C)(C)C
t879	PC (40:6)	FYVNIFOYDIIODX-KNKJIUSSSA-N	24778900	CCCCCC/C=C\CCCCCCCCCC(=O)OC[C@H](COP(=O)([O-])OCC[N+](C)(C)C)OC(=O)CCCCC/C=C\C/C=C\C/C=C\C/C=C\C/C=C\CC
t234	PC (40:6) B	TYRTWVKQVGNGSZ-RGBTVBCDSA-N	52923195	CCCCC/C=C\C/C=C\CCCCCCCCCC(=O)OC[C@H](COP(=O)([O-])OCC[N+](C)(C)C)OC(=O)CCC/C=C\C/C=C\C/C=C\C/C=C\CCCCC
t560	PC (40:7)	BPUROMFCPFGBOT-ZEGPSQTJSA-N	24778982	CCCCC/C=C\C/C=C\CCCCCCCC(=O)OC[C@H](COP(=O)([O-])OCC[N+](C)(C)C)OC(=O)CC/C=C\C/C=C\C/C=C\C/C=C\C/C=C\CCCCC
t561	PC (40:8)	BFCSBEFTXQRIOJ-IMYLGOOQSA-N	53479093	CC/C=C\C/C=C\C/C=C\C/C=C\CCCCCCC(=O)OC[C@H](COP(=O)([O-])OCC[N+](C)(C)C)OC(=O)CCCCCC/C=C\C/C=C\C/C=C\C/C=C\CC
t237	PC (42:10)	GILJCAGAMFVHNE-QEOOLSSISA-N	53479133	CCCCC/C=C\C/C=C\C/C=C\C/C=C\C/C=C\CCC(=O)O[C@H](COC(=O)CCC/C=C\C/C=C\C/C=C\C/C=C\C/C=C\CC)COP(=O)([O-])OCC[N+](C)(C)C
t238	PC (42:5)	APYSSUSAYQRESE-VZWUYPTESA-N	52923591	CCCCC/C=C\C/C=C\CCCCCCCCCCCC(=O)OC[C@H](COP(=O)([O-])OCC[N+](C)(C)C)OC(=O)CCCCCC/C=C\C/C=C\C/C=C\CCCCC
t239	PC (42:6)	DSVRMAGYENFTLY-GDDYDVMSSA-N	52923651	CCCCC/C=C\C/C=C\CCCCCCCCCC(=O)O[C@H](COC(=O)CCCCC/C=C\C/C=C\C/C=C\C/C=C\CCCCC)COP(=O)([O-])OCC[N+](C)(C)C
t562	PC (o-32:0)	SVWBXNAUENUONE-LDLOPFEMSA-N	173570	CCCCCCCCCCCCCCCCO[C@H](COC(=O)CCCCCCCCCCCCCCC)COP(=O)([O-])OCC[N+](C)(C)C
t241	PC (o-34:0)	ZKTXOJMFIAILJG-VQJSHJPSSA-N	24779361	CCCCCCCCCCCCCCCCCC(=O)OC[C@H](COP(=O)([O-])OCC[N+](C)(C)C)OCCCCCCCCCCCCCCCC
t563	PC (p-32:0) or PC (o-32:1)	KEVGQWGZKKFGDC-JCUPVDEDSA-N	53478671	CCCCCCCCCCCCCCCC(=O)OCC(COP(=O)([O-])OCC[N+](C)(C)C)O/C=C\CCCCCCCCCCCCCC
t243	PC (p-32:1) or PC (o-32:2)	FZMYLOBGNYZPQO-QLSONYGBSA-N	52923882	CCCCCCCCCCCCCC/C=C\OC[C@H](COP(=O)([O-])OCC[N+](C)(C)C)OC(=O)CCCCCCC/C=C\CCCCCC

t564	PC (p-34:0) or PC (o-34:1)	QCGUXAIDEOWPBV-SNKLRXETSA-N	53481719	CCCCCCCCCCCCCCCC(=O)O[C@H](COCCCCCCCCCC/C=C\CCCCCC)COP(=O)([O-])OCC[N+](C)(C)C
t566	PC (p-34:1) or PC (o-34:2)	MBRHHFWRXQYYAN-RTVLTNFHSA-N	53480735	CCCCCCCCCCCCCCCC(=O)OC(CO/C=C\CCCCCCCC/C=C\CCCCCC)COP(=O)([O-])OCC[N+](C)(C)C
t245	PC (p-34:1) or PC (o-34:2) A	MBRHHFWRXQYYAN-JEPFLRBFSA-N	70698781	CCCCCCCCCCCCCCCC(=O)O[C@H](CO/C=C\CCCCCCCC/C=C\CCCCCC)COP(=O)([O-])OCC[N+](C)(C)C
t246	PC (p-34:1) or PC (o-34:2) B	KMNVIRCHUMQGHD-RCINKDPXSA-N	52923934	CCCCCCCCCCCCCCCC/C=C\OC[C@H](COP(=O)([O-])OCC[N+](C)(C)C)OC(=O)CCCCCCC/C=C\CCCCCC
t567	PC (p-34:2) or PC (o-34:3)	QLEHHUPUHJPURI-PWYDUFMYSA-N	24779386	CCCCCCCCCCCCCC/C=C\OC[C@H](COP(=O)([O-])OCC[N+](C)(C)C)OC(=O)CCCCCCC/C=C\C/C=C\CCCCC
t568	PC (p-36:1) or PC (o-36:2)	ZOTYCHIFTCFAHC-KOUVQCMKSA-N	53480797	CCCCCCCCCCCCCCCCCC(=O)O[C@H](CO/C=C\CCCCCC/C=C\CCCCCCCC)COP(=O)([O-])OCC[N+](C)(C)C
t249	PC (p-36:1) or PC (o-36:2) B	ZYLPVUZBZNMVMR-ZBBHDILGSA-N	52923754	CCCCCCCCCCCCCCCCCOC[C@H](COP(=O)([O-])OCC[N+](C)(C)C)OC(=O)CCCCCCCCC/C=C\C/C=C\CCCCC
t250	PC (p-36:2) or PC (o-36:3)	DIHWZUCEXWUHOD-IIVNATNGSA-N	53480801	CCCCCCCC/C=C\CCCCCCCC(=O)O[C@H](CO/C=C\CCCCCC/C=C\CCCCCCCC)COP(=O)([O-])OCC[N+](C)(C)C
t570	PC (p-36:3) or PC (o-36:4)	SOUZQPFUXRVDGK-KCTKZSJBSA-N	53481701	CCCCCCCCCCCCCCCCOC[C@H](COP(=O)([O-])OCC[N+](C)(C)C)OC(=O)CCCCCC/C=C\C/C=C\C/C=C\C/C=C\CC
t880	PC (p-36:4) or PC (o-36:5)	IOYKZPNDXIIXLN-LOQSCQKMSA-N	24779388	CCCCCCCCCCCCCC/C=C\OC[C@H](COP(=O)([O-])OCC[N+](C)(C)C)OC(=O)CCC/C=C\C/C=C\C/C=C\C/C=C\CCCCC
t571	PC (p-38:3) or PC (o-38:4)	GWBOVQHRCURSPU-QMFAPAEZSA-N	53480815	CCCCCCCC/C=C\CCCCCC/C=C\OC[C@H](COP(=O)([O-])OCC[N+](C)(C)C)OC(=O)CCCCCCCCC/C=C\C/C=C\CCCCC
t572	PC (p-38:4) or PC (o-38:5)	DBQMOXDLWKVKKG-REWQMPQJSA-N	53480761	CCCCCC/C=C\CCCCCCCC/C=C\OC[C@H](COP(=O)([O-])OCC[N+](C)(C)C)OC(=O)CCCCCC/C=C\C/C=C\C/C=C\CCCCC
t255	PC (p-38:4) or PC (o-38:5) A	YPAPIRJFGNBODV-AMFPDOHCSA-N	53480715	CCCCCCCCCCCCCCCC/C=C\OCC(COP(=O)([O-])OCC[N+](C)(C)C)OC(=O)CCCCCC/C=C\C/C=C\C/C=C\C/C=C\CC
t256	PC (p-38:4) or PC (o-38:5) B	DYFGXBAIDOATAE-DSLLYOFSSA-N	53480759	CCCCCCCC/C=C\C/C=C\C/C=C\CCCC(=O)OC(CO/C=C\CCCCCCCC/C=C\CCCCCC)COP(=O)([O-])OCC[N+](C)(C)C
t573	PC (p-38:5) or PC (o-38:6)	ATTCDOPAYPGSLE-LQULQHAGSA-N	53479121	CCCCCCCC/C=C\CCCCCC/C=C\OC(COC(=O)CCCCCC/C=C\C/C=C\C/C=C\C/C=C\CC)COP(=O)([O-])OCC[N+](C)(C)C
t257	PC (p-38:5) or PC (o-38:6) A	FAKYQMLQEAQOLK-LHZZQLRFSA-N	53480695	CCCCCCCCCCCCCC/C=C\OC[C@H](COP(=O)([O-])OCC[N+](C)(C)C)OC(=O)CC/C=C\C/C=C\C/C=C\C/C=C\C/C=C\CCCCC
t574	PC (p-40:1) or PC (o-40:2)	KYEGAYPFBCAHDL-SPACVREBSA-N	53480827	CCCCCCCCCCCCCCCCCCCCCC(=O)O[C@H](CO/C=C\CCCCCC/C=C\CCCCCCCC)COP(=O)([O-])OCC[N+](C)(C)C
t261	PC (p-40:3) or PC (o-40:4)	RDNHPNJCALITSY-MBZSPAKGSA-N	52923852	CCCCCCCCCCCCCCCCCCCCCCOC[C@H](COP(=O)([O-])OCC[N+](C)(C)C)OC(=O)CCC/C=C\C/C=C\C/C=C\C/C=C\CCCCC
t262	PC (p-40:4) or PC (o-40:5)	UWNFEVACEPZILS-RNNLSGHUSA-N	52924022	CCCCCCCCCCCCCCCCCC/C=C\OC[C@H](COP(=O)([O-])OCC[N+](C)(C)C)OC(=O)CCC/C=C\C/C=C\C/C=C\C/C=C\CCCCC

t263	PC (p-40:5) or PC (o-40:6)	KUHMJRMPHBRAMY-DDURNVNNSA-N	53480775	CCCCCC/C=C\CCCCCCCC/C=C\OCC(COP(=O)([O-])OCC[N+](C)(C)C)OC(=O)CCCCC/C=C\C/C=C\C/C=C\C/C=C\CCCCC
t264	PC (p-40:6) or PC (o-40:7) A	XUVCLJCZWPTAIO-OAKHYACESA-N	53479405	CCCCCCCC/C=C\CCCCCC/C=C\OC(COC(=O)CCCCC/C=C\C/C=C\C/C=C\C/C=C\C/C=C\CC)COP(=O)([O-])OCC[N+](C)(C)C
t265	PC (p-40:6) or PC (o-40:7) B	FMBYBTSZVHUJMV-DIVFMYBRSA-N	53479425	CCCCCCCCCCCCCCCC/C=C\OC(COC(=O)CC/C=C\C/C=C\C/C=C\C/C=C\C/C=C\C/C=C\CC)COP(=O)([O-])OCC[N+](C)(C)C
t267	PC (p-42:2) or PC (o-42:3)	ZLQCRABYIDJNLT-AZPQEANBSA-N	53479549	CCCCCCCC/C=C\CCCCCCCCCCCCCC(=O)OCC(COP(=O)([O-])OCC[N+](C)(C)C)O/C=C\CCCCCC/C=C\CCCCCCCC
t269	PC (p-42:4) or PC (o-42:5)	NLEDXBSUDVLSEN-UFFJXODHSA-N	52924034	CCCCCCCCCCCCCCCCCC/C=C\OC[C@H](COP(=O)([O-])OCC[N+](C)(C)C)OC(=O)CCCCC/C=C\C/C=C\C/C=C\C/C=C\CCCCC
t270	PC (p-42:5) or PC (o-42:6) A	QZMFOSCFDWPISC-KQHSIIGPSA-N	53481769	CCCCC/C=C\C/C=C\C/C=C\CCCCCCCCCOC[C@H](COP(=O)([O-])OCC[N+](C)(C)C)OC(=O)CCCCCCCC/C=C\C/C=C\C/C=C\CCCCC
t271	PC (p-42:5) or PC (o-42:6) B	CMUBJJYJAQLOOD-XSOFIKLRSA-N	52923864	CCCCCCCCCCCCCCCCCCCCOC[C@H](COP(=O)([O-])OCC[N+](C)(C)C)OC(=O)CC/C=C\C/C=C\C/C=C\C/C=C\C/C=C\C/C=C\CC
t272	PC (p-44:4) or PC (o-44:5)	CEZAZXUWDFPTTE-FHIJIHMSSA-N	53481767	CCCCC/C=C\C/C=C\CCCCCCCCCCCCCOC[C@H](COP(=O)([O-])OCC[N+](C)(C)C)OC(=O)CCCCCCCC/C=C\C/C=C\C/C=C\CCCCC
t274	PE (34:2)	HBZNVZIRJWODIB-NHCUFCNUSA-N	46891780	CCCCCCCCCCCCCCCC(=O)OC[C@H](COP(=O)([O-])OCC[NH3+])OC(=O)CCCCCCC/C=C\C/C=C\CCCCC
t275	PE (36:1)	JQKOHRZNEOQNJE-ZZEZOPTASA-N	25244969	CCCCCCCCCCCCCCCCCC(=O)OCC(COP(=O)([O-])OCC[NH3+])OC(=O)CCCCCCC/C=C\CCCCCCCC
t276	PE (36:4)	KZLUVTCXBFEIFJ-XGLJQOENSA-N	52924904	CCCCCCCCCCCCCCCCCC(=O)OC[C@H](COP(=O)(O)OCCN)OC(=O)CCCC/C=C\C/C=C\C/C=C\C/C=C\CC
t277	PE (38:4)	ANRKEHNWXKCXDB-BHFWLYLHSA-N	46891781	CCCCCCCCCCCCCCCCCC(=O)OC[C@H](COP(=O)([O-])OCC[NH3+])OC(=O)CCC/C=C\C/C=C\C/C=C\C/C=C\CCCCC
t278	PE (38:6)	LFGBKOUQHCWBQI-BZGLIJSBSA-N	52924893	CCCCC/C=C\C/C=C\CCCCCCCC(=O)OC[C@H](COP(=O)(O)OCCN)OC(=O)CCC/C=C\C/C=C\C/C=C\C/C=C\CCCCC
t279	PE (p-34:1) or PE (o-34:2)	SMPXBIVJXNXOAL-PRWZWGSOSA-N	53479657	CCCCCCCCCCCCCC/C=C\O[C@H](COC(=O)CCCCCCC/C=C\CCCCCCCC)COP(=O)(O)OCCN
t281	PE (p-36:2) or PE (o-36:3)	CFANDHZPOSNKNO-UDHSZFGOSA-N	53480897	CCCCCCCC/C=C\CCCCCCCC(=O)O[C@H](CO/C=C\CCCCCC/C=C\CCCCCCCC)COP(=O)(O)OCCN
t282	PE (p-36:4) or PE (o-36:5)	ADWDFBQPQIEGRZ-XBICFDGKSA-N	53480870	CCCCCC/C=C\CCCCCCCC/C=C\OC[C@H](COP(=O)(O)OCCN)OC(=O)CCCC/C=C\C/C=C\C/C=C\CCCCC
t283	PE (p-38:4) or PE (o-38:5)	ZTZQZGHJLWFLFQ-VZBWJDOASA-N	53480855	CCCCCCCCCCCCCCCC/C=C\OC[C@H](COP(=O)(O)OCCN)OC(=O)CCCCCC/C=C\C/C=C\C/C=C\C/C=C\CC
t284	PE (p-38:5) or PE (o-38:6)	IQSPCSIULMCRPM-ZAJUHDLGSA-N	53479831	CCCCCCCC/C=C\CCCCCC/C=C\O[C@H](COC(=O)CCCCCC/C=C\C/C=C\C/C=C\C/C=C\CC)COP(=O)(O)OCCN
t286	PE (p-40:5) or PE (o-40:6)	HHQFKPJXVYWLLJ-ABYSKWQHSA-N	53480857	CCCCCCCCCCCCCCCC/C=C\OC[C@H](COP(=O)(O)OCCN)OC(=O)CC/C=C\C/C=C\C/C=C\C/C=C\C/C=C\CCCCC

t25	pelargonic acid	FBUKVWPVBMHYJY-UHFFFAOYSA-N	8158	CCCCCCCCC(=O)O
t24	phenylalanine	COLNVLDHVKWLRT-QMMMGPOBSA-N	6140	C1=CC=C(C=C1)C[C@@H](C(=O)O)N
t23	phosphate	NBIIXXVUZAFLBC-UHFFFAOYSA-N	1004	OP(=O)(O)O
t22	proline	ONIBWKKTOPOVIA-BYPYZUCNSA-N	145742	C1C[C@H](NC1)C(=O)O
t21	pseudo-uridine	PTJWIQPHWPFNBW-GBNDHIKLSA-N	15047	C1=C(C(=O)NC(=O)N1)[C@H]2[C@@H]([C@@H]([C@H](O2)CO)O)O
t19	salicylaldehyde	SMQUZDBALVYZAC-UHFFFAOYSA-N	6998	C1=CC=C(C(=C1)C=O)O
t18	serine	MTCFGRXMJLQNBG-REOHCLBHSA-N	5951	C([C@@H](C(=O)O)N)O
t287	SM (d30:1)	HZCLJRFPXMKWHR-FEBLJDHQSA-N	44260123	CCCCCCCCCCCCC/C=C/[C@H]([C@H](COP(=O)([O-])OCC[N+](C)(C)C)NC(=O)CCCCCCCCCCC)O
t288	SM (d32:0)	MJAFYELZQYPMQG-MPQUPPDSSA-N	44260138	CCCCCCCCCCCCCCC[C@H]([C@H](COP(=O)([O-])OCC[N+](C)(C)C)NC(=O)CCCCCCCCCCCCC)O
t289	SM (d32:1)	KYICBZWZQPCUMO-PSALXKTOSA-N	11433862	CCCCCCCCCCCCC/C=C/[C@H]([C@H](COP(=O)([O-])OCC[N+](C)(C)C)NC(=O)CCCCCCCCCCCCC)O
t291	SM (d33:1)	LQINJRUGTUOHGS-YPDYIYJKSA-N	52931139	CCCCCCCCCCCCCCC(=O)N[C@@H](COP(=O)([O-])OCC[N+](C)(C)C)[C@@H](/C=C/CCCCCCCCCCCCC)O
t292	SM (d34:0)	QHZIGNLCLJPLCU-QPPIDDCLSA-N	9939965	CCCCCCCCCCCCCCC[C@H]([C@H](COP(=O)([O-])OCC[N+](C)(C)C)NC(=O)CCCCCCCCCCCCCCC)O
t293	SM (d34:1)	RWKUXQNLWDTSLO-GWQJGLRPSA-N	9939941	CCCCCCCCCCCCCCCC(=O)N[C@@H](COP(=O)([O-])OCC[N+](C)(C)C)[C@@H](/C=C/CCCCCCCCCCCCC)O
t294	SM (d34:2)	YLWSJLLZUHSIEA-CKSUKHGVSA-N	52931235	CCCCCCCCCCCCCCCC(=O)N[C@@H](COP(=O)([O-])OCC[N+](C)(C)C)[C@@H](/C=C/CCCCCCCC/C=C\CCC)O
t295	SM (d36:0)	JCELSEVNSMXGKA-IOLBBIBUSA-N	44260130	CCCCCCCCCCCCCCCCCC(=O)N[C@@H](COP(=O)([O-])OCC[N+](C)(C)C)[C@@H](CCCCCCCCCCCCCCC)O
t296	SM (d36:1)	LKQLRGMMMAHREN-YJFXYUILSA-N	6453725	CCCCCCCCCCCCCCCCCC(=O)N[C@@H](COP(=O)([O-])OCC[N+](C)(C)C)[C@@H](/C=C/CCCCCCCCCCCCC)O
t297	SM (d36:2)	NBEADXWAAWCCDG-QDDWGVBQSA-N	6443882	CCCCCCCCCCCCC/C=C/[C@H]([C@H](COP(=O)([O-])OCC[N+](C)(C)C)NC(=O)CCCCCCC/C=C\CCCCCCCC)O
t298	SM (d36:3)	YMTVMVYOUDDTQJ-UOMMIRHQSA-N	52931155	CCCCCCCC/C=C\CCCCCCCC(=O)N[C@@H](COP(=O)([O-])OCC[N+](C)(C)C)[C@@H](/C=C/CCCCCCCC/C=C\CCC)O
t301	SM (d38:1)	AADLTHQNYQJHQV-SVLGDMRNSA-N	44260124	CCCCCCCCCCCCCCCCCCCC(=O)N[C@@H](COP(=O)([O-])OCC[N+](C)(C)C)[C@@H](/C=C/CCCCCCCCCCCCC)O

t302	SM (d38:2)	MDRFMTLYKHBJTF-NQYLGBTJSA-N	52931179	CCCCCCCCCCCCC/C=C/[C@H]([C@H](COP(=O)([O-])OCC[N+](C)(C)C)NC(=O)CCCCCCCCC/C=C\CCCCCCCC)O
t305	SM (d40:0)	FONAXCRWZQFJHY-JCGOJSMZSA-N	44260132	CCCCCCCCCCCCCCCCCCCCCCC(=O)N[C@@H](COP(=O)([O-])OCC[N+](C)(C)C)[C@@H](CCCCCCCCCCCCCCC)O
t307	SM (d40:2)	FOULCGVQZYQEQM-DNXGLLHMSA-N	52931201	CCCCCCCCCCC/C=C/[C@H]([C@H](COP(=O)([O-])OCC[N+](C)(C)C)NC(=O)CCCCCCCCCCCCC/C=C\CCCCCCCC)O
t309	SM (d41:1)	SXZWBNWTCVLZJN-NMIJJABPSA-N	46891684	CCCCCCCCCCCCCCCCCCCCCCC(=O)N[C@@H](COP(=O)([O-])OCC[N+](C)(C)C)[C@@H](/C=C/CCCCCCCCCCCCC)O
t310	SM (d41:2) A	JBDGKEXQKCCQFK-JWQIMADESA-N	52931209	CCCCCCCCCCCCCCCCCCCCCCCC(=O)N[C@@H](COP(=O)([O-])OCC[N+](C)(C)C)[C@@H](/C=C/CCCCCCCC/C=C\CCC)O
t312	SM (d42:1)	QEDPUVGSSDPBMD-XTAIVQBESA-N	44260127	CCCCCCCCCCCCCCCCCCCCCCCC(=O)N[C@@H](COP(=O)([O-])OCC[N+](C)(C)C)[C@@H](/C=C/CCCCCCCCCCCCC)O
t314	SM (d42:2)	DACOGJMBYLZYDH-GXJPFUDISA-N	52931217	CCCCCCCCCCCCCCCCCCCCCCCCC(=O)N[C@@H](COP(=O)([O-])OCC[N+](C)(C)C)[C@@H](/C=C/CCCCCCCC/C=C\CCC)O
t315	SM (d42:3)	TXFLWJQVQCDUDZ-BRUGZULGSA-N	52931215	CCCCCCCC/C=C\CCCCCCCCCCCCCC(=O)N[C@@H](COP(=O)([O-])OCC[N+](C)(C)C)[C@@H](/C=C/CCCCCCCC/C=C\CCC)O
t316	SM (d43:1)	LXMARZYBSFYVSY-KUQVZNNZSA-N	52931225	CCCCCCCCCCCCCCCC[C@H]([C@H](COP(=O)([O-])OCC[N+](C)(C)C)NC(=O)CCCCCCCCCCCCC/C=C\CCCCCCCC)O
t16	succinic acid	KDYFGRWQOYBRFD-UHFFFAOYSA-N	1110	C(CC(=O)O)C(=O)O
t15	taurine	XOAAWQZATWQOTB-UHFFFAOYSA-N	1123	C(CS(=O)(=O)O)N
t457	TG (48:0)	PVNIQBQSYATKKL-UHFFFAOYSA-N	11147	CCCCCCCCCCCCCCCC(=O)OCC(COC(=O)CCCCCCCCCCCCCCC)OC(=O)CCCCCCCCCCCCCCC
t458	TG (48:1)	FEKLSEFRUGWUOS-DLOIZKPKSA-N	9543986	CCCCCCCCCCCCCCCC(=O)OC[C@H](COC(=O)CCCCCCC/C=C\CCCCCC)OC(=O)CCCCCCCCCCCCCCC
t459	TG (48:2)	RUOVJPPUXXFZPC-YZEIBMOJSA-N	9543987	CCCCCCCCCCCCCCCC(=O)OC[C@H](COC(=O)CCCCCCC/C=C\CCCCCC)OC(=O)CCCCCCC/C=C\CCCCCC
t320	TG (49:0)	TTWJTJMWHOYBPQ-ANFMRNGASA-N	9543988	CCCCCCCCCCCCCCCCC(=O)OC[C@@H](COC(=O)CCCCCCCCCCCCCCC)OC(=O)CCCCCCCCCCCCCCC
t460	TG (49:1)	VYYGQDOPVVYUKW-UKFBYESTSA-N	9543991	CCCCCCCCCCCCCCCC(=O)OC[C@H](COC(=O)CCCCCCC/C=C\CCCCCCC)OC(=O)CCCCCCCCCCCCCCC
t322	TG (49:2)	QZYSUBAQYSVFNX-PSMULLBHSA-N	9543993	CCCCCCCCCCCCCCCCC(=O)OC[C@@H](COC(=O)CCCCCCC/C=C\CCCCCC)OC(=O)CCCCCCC/C=C\CCCCCC
t461	TG (49:3)	DIGMYZZFQSIQBD-PNLKURBTSA-N	56938088	CCCCCCCCCCCCCC(=O)OC[C@H](COC(=O)CCCCCCC/C=C\C/C=C\CCCCC)OC(=O)CCCCCCC/C=C\CCCCCCC
t462	TG (50:0)	MARPCPMDFOPPJX-UHFFFAOYSA-N	545588	CCCCCCCCCCCCCCCCC(=O)OCC(COC(=O)CCCCCCCCCCCCCCCC)OC(=O)CCCCCCCCCCCCC

t463	TG (50:1)	YHMDGPZOSGBQRH-YYSBDVFPSA-N	25240460	CCCCCCCCCCCCCCCC(=O)OC[C@H](COC(=O)CCCCCCC/C=C\CCCCCCC)OC(=O)CCCCCCCCCCCCCCC
t464	TG (50:2)	QEZWFCZNHWUARW-XQCAQTCHSA-N	9544010	CCCCCCCCCCCCCCCCCC(=O)OC[C@@H](COC(=O)CCCCCCC/C=C\CCCCCC)OC(=O)CCCCCCC/C=C\CCCCCC
t465	TG (50:3)	UFHNZOACKFBCOM-YXKNDSBASA-N	25240357	CCCCCCCC/C=C\CCCCCCCC(=O)OC[C@@H](COC(=O)CCCCCCC/C=C\CCCCCC)OC(=O)CCCCCCC/C=C\CCCCCC
t466	TG (50:4)	PVMBAGXWHHZKFP-JMPJWMFJSA-N	25240359	CCCCCC/C=C\CCCCCCCC(=O)OC[C@H](COC(=O)CCCCCCC/C=C\C/C=C\CCCCC)OC(=O)CCCCCCC/C=C\CCCCCC
t467	TG (50:5)	AFTBPUXZTDLRSP-UDQIKIEDSA-N	9544045	CCCCCC/C=C\CCCCCCCC(=O)OC[C@H](COC(=O)CCCCCCC/C=C\C/C=C\C/C=C\CC)OC(=O)CCCCCCC/C=C\CCCCCC
t468	TG (51:1)	OZAXLAGNPZMZAD-BOEMPQCLSA-N	9544006	CCCCCCCCCCCCCCCCC(=O)OC[C@H](COC(=O)CCCCCCC/C=C\CCCCCCC)OC(=O)CCCCCCCCCCCCCCCC
t469	TG (51:2)	NSNSZGBCOIKUBU-SZOKBDNISA-N	9544013	CCCCCCCCCCCCCCCCC(=O)OC[C@H](COC(=O)CCCCCCC/C=C\CCCCCCC)OC(=O)CCCCCCC/C=C\CCCCCCC
t470	TG (51:3)	ISSGPXMQOMAFMJ-DMGKHJLRSA-N	9544023	CCCCCCC/C=C\CCCCCCCC(=O)OCC(OC(=O)CCCCCCC/C=C\CCCCCCC)COC(=O)CCCCCCC/C=C\CCCCCCC
t471	TG (51:4)	IIRQXNVLAXQEKB-KBEZCZBDSA-N	9544052	CCCCCCCC/C=C\CCCCCCCC(=O)OC[C@@H](COC(=O)CCCCCCC/C=C\CCCCCC)OC(=O)CCCCCCC/C=C\C/C=C\CCCC
t472	TG (52:0)	SDNYRTVJOFMYIW-OIVUAWODSA-N	545690	CCCCCCCCCCCCCCCCCC(=O)OC[C@@H](COC(=O)CCCCCCCCCCCCCC)OC(=O)CCCCCCCCCCCCCCCCC
t473	TG (52:1)	NPCZZYKITFKRQZ-RFBIWTDZSA-N	5365005	CCCCCCCCCCCCCCCCCC(=O)OC(COC(=O)CCCCCCCCCCCCCCC)COC(=O)CCCCCCC/C=C\CCCCCCCC
t474	TG (52:2)	TXMWKTABZBAJCW-QLHBUVOUSA-N	56938176	CCCCCCCCCCCCCCCCCCCC(=O)OC[C@@H](COC(=O)CCCCCCCCCCCC)OC(=O)CCCCCCC/C=C\C/C=C\CCCCC
t475	TG (52:3)	DQXQIWIQYYEGLG-MMWLGPPDSA-N	56938177	CCCCCCCCCCCCCC(=O)OC[C@H](COC(=O)CCCCCCCCC/C=C\CCCCCCCC)OC(=O)CCCCCCC/C=C\C/C=C\CCCCC
t476	TG (52:4)	WHSWXEYWNPTUPW-HNJDVRDNSA-N	25240364	CCCCCCCC/C=C\CCCCCCCC(=O)O[C@H](COC(=O)CCCCCCC/C=C\CCCCCC)COC(=O)CCCCCCC/C=C\C/C=C\CCCCC
t477	TG (52:5)	CQZAAIKPSLHIBC-KDJOUNIJSA-N	25240366	CCCCCC/C=C\CCCCCCCC(=O)OC[C@H](COC(=O)CCCCCCC/C=C\C/C=C\CCCCC)OC(=O)CCCCCCC/C=C\C/C=C\CCCCC
t478	TG (52:6)	SSOSFUDNINFYLJ-KIYGNKBKSA-N	56938180	CCCCCCCCCCCCCC(=O)OC[C@H](COC(=O)CCC/C=C\C/C=C\C/C=C\C/C=C\CCCCC)OC(=O)CCCCCCC/C=C\C/C=C\CCCCC
t479	TG (53:2)	RSINITWKVQRWSZ-RFVLVDBCSA-N	9544102	CCCCCCCCCCCCCCCCCC(=O)OC[C@@H](COC(=O)CCCCCCC/C=C\C/C=C\CCCC)OC(=O)CCCCCCCCCCCCCCCCC
t480	TG (53:3)	ZNQBEJJYVJSZLM-LEDQTTRKSA-N	9544126	CCCCCCCC/C=C\CCCCCCCC(=O)OC[C@@H](COC(=O)CCCCCCC/C=C\CCCCCCC)OC(=O)CCCCCCC/C=C\CCCCCCCC
t324	TG (53:4)	BMSDHYZLQWTKSQ-LSJAAEOESA-N	9544152	CCCCCCCC/C=C\CCCCCCCC(=O)OC[C@@H](COC(=O)CCCCCCC/C=C\C/C=C\CCCC)OC(=O)CCCCCCC/C=C\CCCCCCCC

t481	TG (53:5)	QHYAATSKYBYSLG-BXDFBOBBSA-N	9544183	CCCCCCC/C=C\CCCCCCCC(=O)OC[C@H](COC(=O)CCCCCCC/C=C\C/C=C\CCCCC)OC(=O)CCCCCCC/C=C\C/C=C\CCCCC
t483	TG (54:1)	YFFIQXNTTVSKJC-NZEOUKRFSA-N	16058371	CCCCCCCCCCCCCCCCCC(=O)OC[C@H](COC(=O)CCCCCCC/C=C\CCCCCCCC)OC(=O)CCCCCCCCCCCCCCCCC
t484	TG (54:2)	WUUWGORPQFKFQN-XDVOZUNOSA-N	56938183	CCCCCCCCCCCCCCCCCCCCC(=O)OC[C@@H](COC(=O)CCCCCCCCCCCC)OC(=O)CCCCCCC/C=C\C/C=C\CCCCC
t326	TG (54:3)	PHYFQTYBJUILEZ-IUPFWZBJSA-N	5497163	CCCCCCCC/C=C\CCCCCCCC(=O)OCC(OC(=O)CCCCCCC/C=C\CCCCCCCC)COC(=O)CCCCCCC/C=C\CCCCCCCC
t329	TG (54:4)	BRLGHZXETDWABO-NOFIOOQLSA-N	9544255	CCCCCCCCCCCCCCCCCC(=O)OC[C@H](COC(=O)CCCCCCC/C=C\C/C=C\C/C=C\CC)OC(=O)CCCCCCC/C=C\CCCCCCCC
t486	TG (54:5)	OEJXMJPFOHYSIU-GRLFFVHSSA-N	9544294	CCCCCCCC/C=C\CCCCCCCC(=O)OC[C@H](COC(=O)CCCCCCC/C=C\C/C=C\C/C=C\CC)OC(=O)CCCCCCC/C=C\CCCCCCCC
t488	TG (54:6)	CDNDFDKFZBPPFW-AXJGXPKFSA-N	9544363	CCCCCCCC/C=C\CCCCCCCC(=O)O[C@H](COC(=O)CCCCCCC/C=C\CCCCCC)COC(=O)CCC/C=C\C/C=C\C/C=C\C/C=C\CCCCC
t489	TG (54:8)	BMPVTDWOWBNPJU-NYRSPQLFSA-N	9544413	CCCCC/C=C\C/C=C\C/C=C\C/C=C\CCCC(=O)OC[C@@H](COC(=O)CCCCCCC/C=C\C/C=C\CCCC)OC(=O)CCCCCCC/C=C\C/C=C\CCCC
t490	TG (56:2)	PDEQUPGHMOMBFC-FYEHETCMSA-N	9544390	CCCCCCCCCCCCCCCCCCCCC(=O)OC[C@@H](COC(=O)CCCCCCCCCCCCCCCCC)OC(=O)CCCCCCC/C=C\C/C=C\CCCCC
t491	TG (56:3)	QXMHHXQBBKDSSL-BAQZNRHJSA-N	9544447	CCCCCCCC/C=C\CCCCCCCCCC(=O)OC[C@@H](COC(=O)CCCCCCC/C=C\CCCCCCCC)OC(=O)CCCCCCC/C=C\CCCCCCCC
t330	TG (56:4)	YONCDTJKIZDSKQ-IYASBODOSA-N	25240379	CCCCCCCC/C=C\CCCCCCCCCC(=O)OC[C@@H](COC(=O)CCCCCCC/C=C\CCCCCCCC)OC(=O)CCCCCCC/C=C\C/C=C\CCCCC
t493	TG (56:5) A	UHEJWASONFIROS-YPSHDQQVSA-N	25240380	CCCCCCCC/C=C\CCCCCCCCCC(=O)OC[C@@H](COC(=O)CCCCCCC/C=C\C/C=C\CCCCC)OC(=O)CCCCCCC/C=C\C/C=C\CCCCC
t494	TG (56:6)	ZTNDRFCABXFVMY-WJTCTALZSA-N	9544625	CCCCC/C=C\C/C=C\CCCCCCCCCC(=O)OC[C@@H](COC(=O)CCCCCCC/C=C\C/C=C\CCCCC)OC(=O)CCCCCCC/C=C\C/C=C\CCCCC
t332	TG (56:7) B	DODZUDCYRVWEOJ-GKZBLMSTSA-N	9544695	CCCCC/C=C\C/C=C\CCCCCCCC(=O)OC[C@H](COC(=O)CCCCCC/C=C\C/C=C\C/C=C\CCCCC)OC(=O)CCCCCCC/C=C\C/C=C\CCCCC
t496	TG (56:8)	UBGUHMDKBGQUND-VPFWBQFRSA-N	9544762	CCCCCCCCCCCCCCCC(=O)OC[C@H](COC(=O)CCC/C=C\C/C=C\C/C=C\C/C=C\CCCCC)OC(=O)CCC/C=C\C/C=C\C/C=C\C/C=C\CCCCC
t498	TG (58:1)	OWZMHFAFGQCCNI-FBXRAONGSA-N	25240381	CCCCCCCCCCCCCCCCCCCCC(=O)OC[C@@H](COC(=O)CCCCCCC/C=C\CCCCCCCC)OC(=O)CCCCCCCCCCCCCCCCCCC
t497	TG (58:10)	GXWBCAVCOMAOHT-VMCJOIRWSA-N	9545277	CCCCC/C=C\C/C=C\CCCCCCCC(=O)OC[C@H](COC(=O)CCC/C=C\C/C=C\C/C=C\C/C=C\CCCCC)OC(=O)CCC/C=C\C/C=C\C/C=C\C/C=C\CCCCC
t499	TG (58:6)	GSNFRUMSEHHPSY-LCXCSEBNSA-N	9544977	CCCCCCCC/C=C\CCCCCCCCCC(=O)O[C@H](COC(=O)CCCCCCC/C=C\CCCCCCCC)COC(=O)CCC/C=C\C/C=C\C/C=C\C/C=C\CCCCC
t336	TG (58:8)	KWIGMCRWEINBIR-HUPVKWKYSA-N	9545124	CCCCCCCCCCCCCCCCCC(=O)OC[C@H](COC(=O)CCC/C=C\C/C=C\C/C=C\C/C=C\CCCCC)OC(=O)CCC/C=C\C/C=C\C/C=C\C/C=C\CCCCC

t500	TG (58:9)	RVXFSLZMZOFGEQ-SWIIBWKZSA-N	9545200	CCCCCCCC/C=C\CCCCCCCC(=O)OC[C@H](COC(=O)CCC/C=C\C/C=C\C/C=C\C/C=C\CCCCC)OC(=O)CCC/C=C\C/C=C\C/C=C\C/C=C\CCCCC
t14	threitol	UNXHWFMMPAWVPI-QWWZWVQMSA-N	169019	C([C@H]([C@@H](CO)O)O)O
t13	threonic acid	JPIJQSOTBSSVTP-STHAYSLISA-N	5460407	C([C@@H]([C@H](C(=O)O)O)O)O
t12	threonine	AYFVYJQAPQTCCC-GBXIJSLDSA-N	6288	C[C@H]([C@@H](C(=O)O)N)O
t11	tocopherol alpha-	NCYCYZXNIZJOKI-OVSJKPMPSA-N	638015	CC1=C(C(CCC1)(C)C)/C=C/C(=C/C=C/C(=C/C=O)/C)/C
t10	tocopherol gamma-	QUEDXNHFTDJVIY-DQCZWYHMSA-N	92729	CC1=C(C=C2CC[C@@](OC2=C1C)(C)CCC[C@H](C)CCC[C@H](C)CCCC(C)C)O
t9	trans-4-hydroxyproline	PMMYEEVYMWASQN-DMTCNVIQSA-N	5810	C1[C@H](CN[C@@H]1C(=O)O)O
t8	tryptophan	QIVBCDIJIAJPQS-VIFPVBQESA-N	6305	C1=CC=C2C(=C1)C(=CN2)C[C@@H](C(=O)O)N
t7	tyrosine	OUYCCCASQSFEME-QMMMGPOBSA-N	6057	C1=CC(=CC=C1C[C@@H](C(=O)O)N)O
t6	urea	XSQUKJJJFZCRTK-UHFFFAOYSA-N	1176	C(=O)(N)N
t5	uric acid	LEHOTFFKMJEONL-UHFFFAOYSA-N	1175	C12=C(NC(=O)N1)NC(=O)NC2=O
t4	uridine	DRTQHJPVMGBUCF-XVFCMESISA-N	6029	C1=CN(C(=O)NC1=O)[C@H]2[C@@H]([C@@H]([C@H](O2)CO)O)O
t3	valine	KZSNJWFQEVHDMF-BYPYZUCNSA-N	6287	CC(C)[C@@H](C(=O)O)N
t2	xanthine	LRFVTYWOQMYALW-UHFFFAOYSA-N	1188	C1=NC2=C(N1)C(=O)NC(=O)N2
t1	xylulose	LQXVFWRQNMEDEE-PYHARJCCSA-N	439205	C1[C@@H]([C@H](C(O1)(CO)O)O)O

Supplemental Table 2: Proportion and amount of subjects eating any amount of food subgroups among TEDDY mAb+ cases and matched controls with metabolomics at 9 months and seroconversion

	Infancy 9-months (n=530)		
**Food groups (bold) and subgroups**	**n Consumers**	**Mean g/day**	**% Consumers**
**BREAST MILK**	229	345.19	43.2
**CEREALS**			
Gluten-containing cereals (wheat, rye, barley)	469	15.56	88.5
Gluten-free cereals (rice, oats, corn, other GF flours and starches)	524	30.81	98.9
**FRUIT AND BERRIES**			
Apple	364	44.02	68.7
Berries	253	20.63	47.7
Citrus	20	17.59	3.8
Other fresh fruits	484	59.48	91.3
Processed fruits (canned and dried)	268	31.70	50.6
**FRUIT, BERRY, VEGETABLE JUICES**			
Apple Juice	183	22.14	34.5
Berry Juice	8	16.89	1.5
Citrus Juice	149	7.43	28.1
Other fruit juices	205	15.27	38.7
Mixed Fruit, Fruit/Vegetables, Vegetables Juices	74	34.64	14.0
**VEGETABLES**			
Potatoes	416	54.60	78.5
Roots, Sweet Potatoes	488	39.26	92.1
Leafy Vegetables (and Cabbage)	266	19.05	50.2
Fruit Vegetables	391	30.38	73.8
Onions	296	8.17	55.9
Legumes	241	24.85	45.5
Processed Vegetables	237	15.68	44.7
Soy	29	15.15	5.5
**NUTS, SEEDS**	43	0.89	8.1
**FATS AND OILS**			
Vegetables Oils	432	7.99	81.5
Saturated Fats (solid vegetable, animal fats)	251	2.13	47.4
Low-Fat Butter/Margarine	42	4.83	7.9
High-Fat Butter/Margarine	163	3.62	30.8
**MILK AND MILK PRODUCTS**			

Fat-free milk	252	58.40	47.6
Milk	195	224.12	36.8
Creams	124	7.25	23.4
Ice cream	15	10.73	2.8
Sour Milk	193	50.47	36.4
Cheese	178	9.42	33.6
**NON-DAIRY PRODUCTS**	2	13.00	0.4
**MEAT AND MEAT PRODUCTS**			
Red Meat	368	26.39	69.4
Poultry	300	19.50	56.6
Processed Meats	67	22.10	12.6
**FISH AND FISH PRODUCTS**	134	18.67	25.3
**EGGS**	102	8.08	19.3
**BEVERAGES**			
Other Beverages	32	75.84	6.0
Sugar-Sweetened Beverages	90	39.77	17.0
Alcohol	0	.	0.0
**CONFECTIONARY**	10	11.27	1.9
**INFANT FORMULAS**	259	523.18	48.9
**MISCELLANEOUS**	10	6.45	1.9

Supplemental Table 3: Characteristics and outcomes of TEDDY nested case-control 1:3 matched study at seroconversion

	**Outcomes**			
	**IA**	**IAA**	**GADA**	**mAb+**
**Matching Characteristic**	**n (%)**	**n (%)**	**n (%)**	**n (%)**
**Clinical Center**				
Colorado	51 (14.5)	22 (12.9)	17 (15.0)	30 (14.2)
Georgia	28 (8.0)	8 (4.7)	12 (10.6)	18 (8.5)
Washington	32 (9.1)	16 (9.4)	14 (12.4)	15 (7.1)
Finland	100 (28.4)	59 (34.5)	23 (20.4)	72 (34.1)
Germany	28 (8.0)	11 (6.4)	9 (8.0)	16 (7.6)
Sweden	113 (32.1)	55 (32.2)	38 (33.6)	60 (28.4)
**Sex**				
Female	158 (44.9)	71 (41.5)	56 (49.6)	91 (43.1)
Male	194 (55.1)	100 (58.5)	57 (50.4)	120 (56.9)
**FDR/GP Status**				
First Degree Relative	79 (22.4)	37 (21.6)	27 (23.9)	53 (25.1)
General Population	273 (77.6)	134 (78.4)	86 (76.1)	158 (74.9)
**Long Distance Protocol**				
Yes	43 (12.2)	17 (9.9)	16 (14.2)	24 (11.4)
No	309 (87.8)	154 (90.1)	97 (85.8)	187 (88.6)

Supplemental Figure 2: **Volcano plots of the association between 853 metabolites measured at seroconversion, pre-seroconversion, and 9-months and development of islet autoimmunity (IA), multiple autoantibody positivity (mAb+), IAA, and GADA in the TEDDY study.**

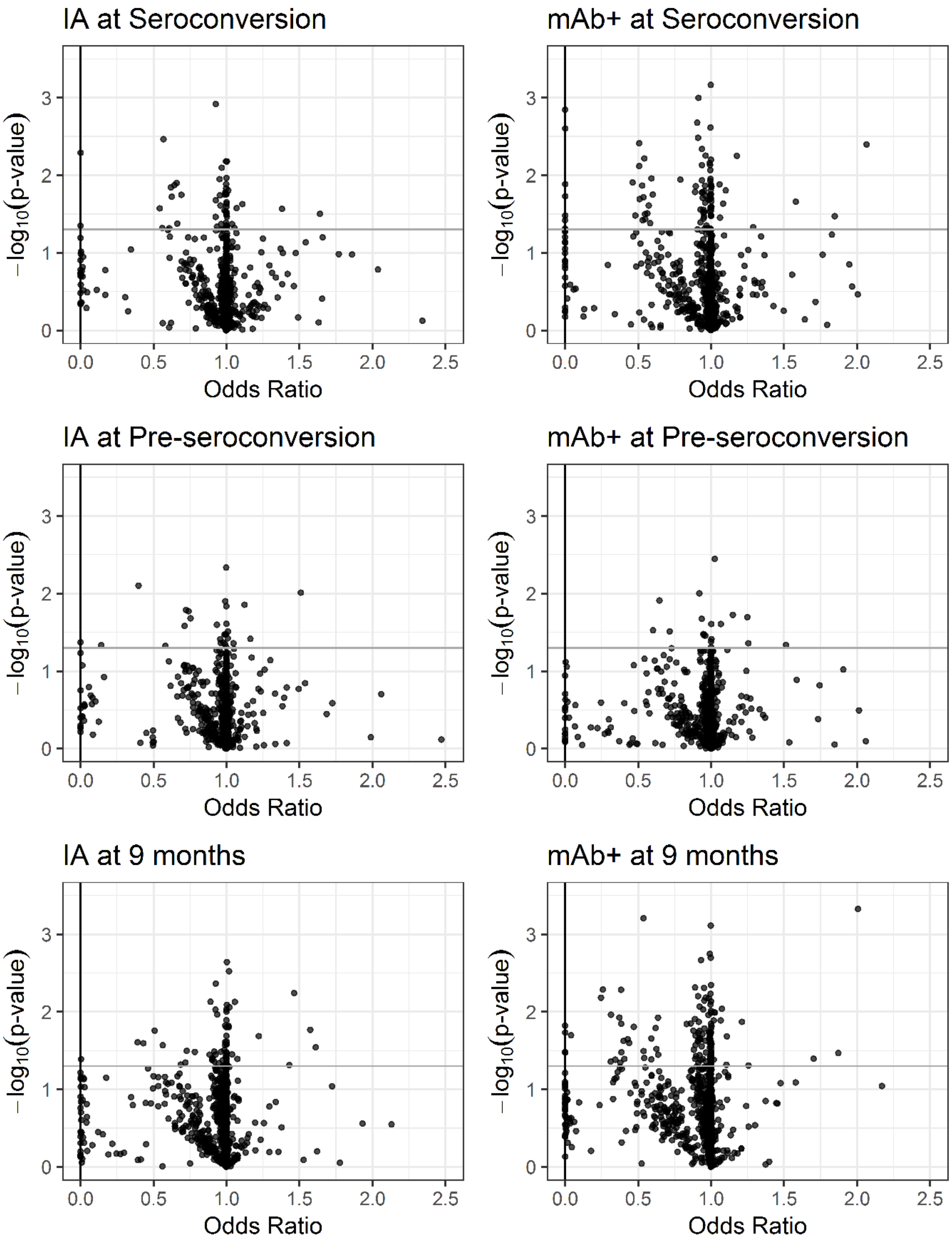

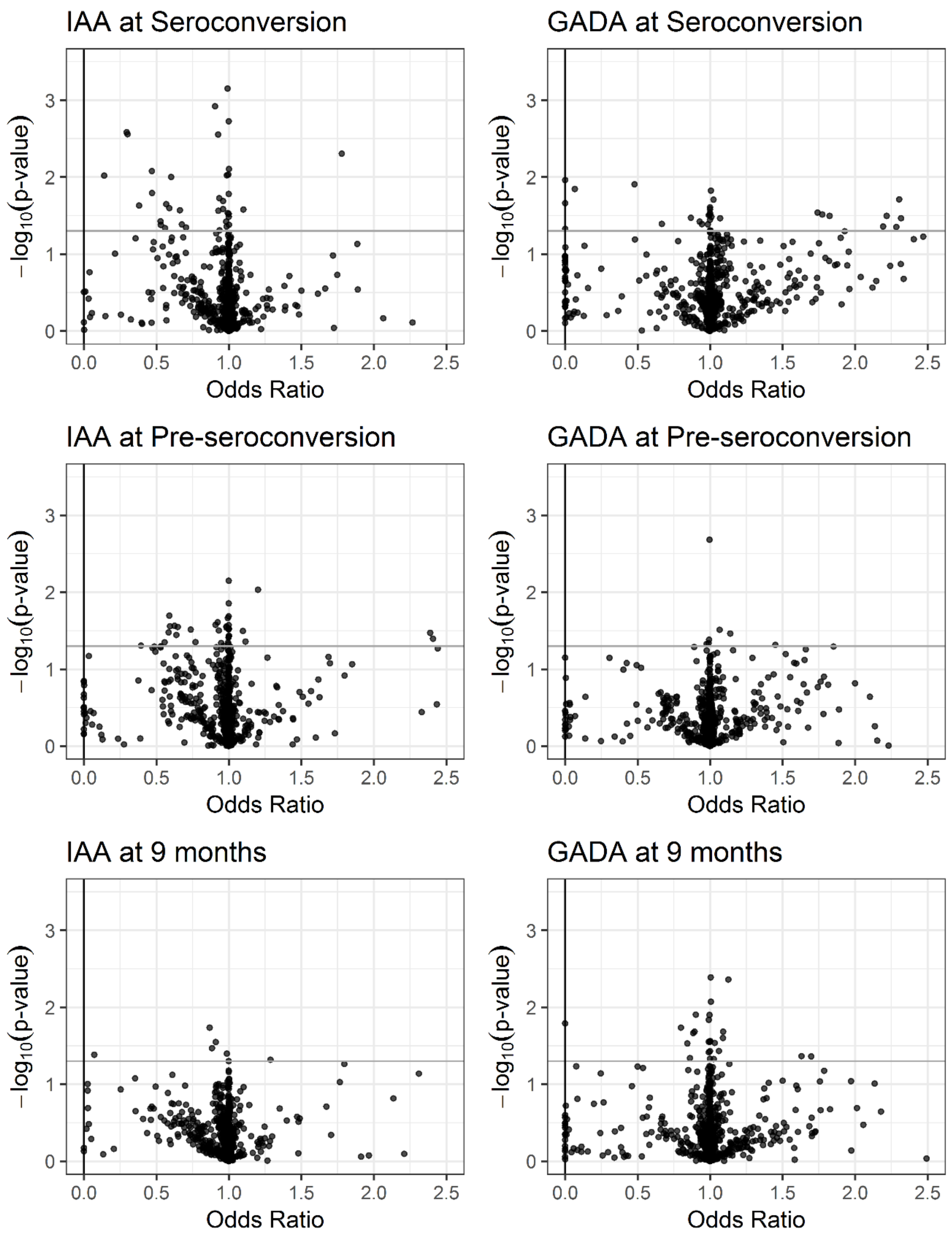
IAA at Seroconversion
GADA at Seroconversion
IAA at Pre-seroconversion
GADA at Pre-seroconversion
IAA at 9 months
GADA at 9 months
$-\log_{10}$(p-value)
Odds Ratio
0
1
2
3
0.0
0.5
1.0
1.5
2.0
2.5

Supplemental Table 4: ChemRICH summary of chemically similar metabolite groups significantly associated with mAb+ (nominal p-value for group<0.05)

		Seroconversion				Pre-seroconversion				Infancy			
**Metabolite Groups**		**Metabolite**		**Group**		**Metabolite**		**Group**		**Metabolite**		**Group**	
**Name**	**Total p**	**p, p<0.05**	**p, OR<1**	**p-value**	**q-value**	**p, p<0.05**	**p, OR<1**	**p-value**	**q-value**	**p, p<0.05**	**p, OR<1**	**p-value**	**q-value**
Unsaturated Phosphatidylcholines	76	5	3	2.1E-05	0.0003	2	1	0.013	0.35	29	28	2.2E-20	6.6E-19
Sphingomyelins	16									10	10	1.1E-08	1.6E-07
Phosphatidylethanolamines	10	3	3	0.0047	0.042					3	3	2.9E-06	2.9E-05
Galactosylceramides	4									2	2	4.4E-05	0.00033
Dicarboxylic Acids	5									3	0	0.0019	0.012
Phospholipid Ethers	6									2	2	0.0032	0.016
Unsaturated Triglycerides	43	17	15	3.1E-15	9E-14								
Amino Acids	6	2	2	0.0074	0.042								
Saturated FA	7	3	0	0.0074	0.042								
Diglycerides	11	3	3	0.014	0.064								
Amino Acids, Aromatic	4	2	2	0.03	0.12								

ChemRICH inputs: p-value and OR=Odds Ratio from conditional logistic regression models adjusted for HLA (DR3/4) and age at blood draw date
p=number of metabolites

Supplemental Table 5: Correlation between key metabolites and dietary pattern scores in infancy.

	Metabolite-related dietary patterns					
	1		2		3	
	r	p-value	r	p-value	r	p-value
PC (34:3)	0.1275	0.0033	0.07447	0.0871	0.30464	<.0001
SM (d41:2) A	0.0415	0.3408	0.17774	<.0001	-0.07351	0.0912
PE (34:2)	0.38408	<.0001	0.10781	0.0131	-0.02897	0.5062
GlcCer (d41:1)	-0.12619	0.0036	0.25199	<.0001	-0.06429	0.1397
Adipic acid	0.00103	0.9812	-0.01747	0.6884	0.00241	0.9559
PC (p-32:0) or PC (o-32:1)	-0.12968	0.0028	0.22418	<.0001	0.16656	0.0001

r=Pearson correlation coefficient

Supplemental Table 6: Cohort characteristics of TEDDY children with food records at age 9-months

	mAb+ by age 6 yr		mAb- at age 6 yr		
	**n**	**%**	**n**	**%**	**p-value**
Total Subjects	300		6237		
Clinical Center					0.196
Colorado	52	17.33	987	15.82	
Georgia	25	8.33	638	10.23	
Washington	29	9.67	837	13.42	
Finland	77	25.67	1406	22.54	
Germany	23	7.67	365	5.85	
Sweden	94	31.33	2004	32.13	
Female	139	46.33	3075	49.3	0.315
First degree relative	71	23.67	613	9.83	<0.001
HLA-DR3/4	169	56.33	2354	37.74	<0.001
	**Mean**	**SD**	**Mean**	**SD**	**p-value**
Age at first Ab (days)	990.1	517.3	--		
Dietary Pattern Scores					
1	-0.055	1.07	0.004	0.98	0.349
2	-0.076	0.88	0.005	0.93	0.120
3	0.082	1.06	0.008	1.04	0.238
